# Photoactive antimicrobial coating based on a PEDOT-fullerene C_60_ polymeric dyad†

**DOI:** 10.1039/d1ra03417k

**Published:** 2021-07-05

**Authors:** Eugenia Reynoso, Andrés M. Durantini, Claudia A. Solis, Lorena P. Macor, Luis A. Otero, Miguel A. Gervaldo, Edgardo N. Durantini, Daniel A. Heredia

**Affiliations:** IDAS-CONICET, Departamento de Química, Facultad de Ciencias Exactas Físico-Químicas y Naturales, Universidad Nacional de Río Cuarto Agencia Postal Nro. 3 X5804BYA Río Cuarto Córdoba Argentina dheredia@exa.unrc.edu.ar +54 358 76233 +54 358 4676538; IITEMA-CONICET, Departamento de Química, Facultad de Ciencias Exactas Físico-Químicas y Naturales, Universidad Nacional de Río Cuarto Agencia Postal Nro. 3 X5804BYA Río Cuarto Córdoba Argentina

## Abstract

A photostable and photodynamic antimicrobial surface was successfully obtained and applied to photoinactivate microorganisms. This approach was based on the synthesis of a fullerene C_60_ derivative (EDOT-C_60_) where fullerene C_60_ is covalently linked to 3,4-ethylenedioxythiophene (EDOT) through a 1,3-dipolar cycloaddition reaction. This dual-functional monomer bears an EDOT center connected *via* an alkyl chain to a fullerene C_60_ moiety. In this structure, EDOT acts as an electropolymerizable unit that allows the film formation over conducting substrates, while fullerene C_60_ performs the photodynamic antimicrobial activity. Electrochemical polymerization of EDOT was used to obtain stable and photodynamic polymeric films (PEDOT-C_60_) in a controllable procedure. Cyclic voltammetry and UV-visible spectroscopy studies showed that the fullerene C_60_ units were not altered during the electropolymerization process, obtaining surfaces with high fullerene content. Photobleaching measurements demonstrated that the electropolymerized films were highly photostable. Moreover, photodynamic properties of PEDOT-C_60_ were compared with fullerene C_60_ and showed that electrodeposited films were able to generate reactive oxygen species (ROS) through the two photomechanisms, producing singlet molecular oxygen (type II) and superoxide radical anion (type I). All studies demonstrated that fullerene C_60_ moieties covalently attached to the polymeric matrix mainly conserve the photodynamic characteristics. Hence, photodynamic action sensitized by PEDOT-C_60_ was assessed *in vitro* against *Staphylococcus aureus*. The photosensitized inactivation by the electropolymerized films on bacteria suspensions produced >99.9% reduction in *S. aureus* survival. Fluorescence microscopy experiments with *S. aureus* adhered to the PEDOT-C_60_ surface showed a complete microbe annihilation. Also, the eradication of biofilms formed on PEDOT-C_60_ surfaces resulted in a photokilling >99.9% after visible light irradiation. Our results demonstrated that these antimicrobial photodynamic polymeric films are a promising and versatile platform to photoinactivate microorganisms and to obtain photostable self-sterilizing surfaces.

## Abstract 2

A photostable and photodynamic antimicrobial surface was developed. The antimicrobial activity of the material reached outstanding levels of inactivation under different conditions: planktonic suspensions, adhered cells to the surface, and biofilms.

## Graphical abstract

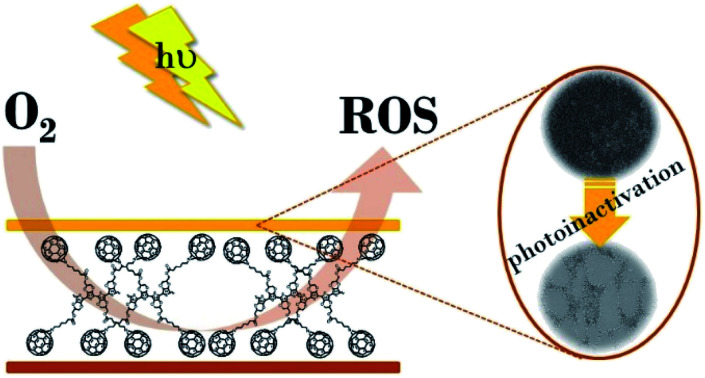

## 1. Introduction

Hospital-acquired infections, also known as nosocomial infections, constitute a significant health problem nowadays.^1^ Not only do they prolong the hospitalization period but also remarkably increase morbidity and mortality in hospitalized patients. Also, the prolonged stay usually requires new antimicrobial treatments and supplementary studies, which results in a substantial increase in healthcare costs.^2^ Consequently, nosocomial infections need to be controlled within healthcare environments.^3^ Infections can be produced by an extensive variety of pathogens;^4^ however, *Staphylococcus aureus* prevails in hospital-associated facilities. This pathogen can colonize and form biofilms on surfaces,^5,6^ spreading resistant strains among healthcare personnel, medical instruments, and patients.^2,5–8^ Therefore, the development of antimicrobial coatings and self-sterilizing surfaces is crucial to diminish the frequency of infections, not only in hospital but also in common use facilities.

The development of self-sterilizing surfaces has become a trending topic in the last years.^9–11^ Aiming to reduce bacterial resistance spread, photodynamic inactivation (PDI) of microorganisms has been purposed as a promising and innovative approach to treat bacterial infections.^12^ This methodology is based on a light-activated photosensitizer (PS) combined with molecular oxygen, O_2_(^3^Σ^−^_g_). The PS excited state, generated by photon absorption, can transfer energy to O_2_(^3^Σ^−^_g_), generating singlet molecular oxygen (O_2_(^1^Δ_g_), type II mechanism), or react directly with biological substrates producing radicals and, finally, other reactive oxygen species (ROS, type I mechanism).^13^ These cytotoxic ROS damage multiple macromolecules and organelles of the cellular system producing irreversible changes, overcoming any defense mechanism activated by the host and leading to the cell death. This therapeutic technique provides a powerful platform for the treatment of broad-spectrum pathogens, even antibiotic resistant bacteria.^14,15^ Furthermore, this antimicrobial strategy has also shown superior outcomes to eradicate biofilms in comparison with other conventional treatments.^16–18^

A wide variety of PSs attached to different surfaces have been employed for the inactivation of pathogenic microorganisms.^19–24^ Thereby, surfaces with immobilized PSs can be used to conserve sterilized and sanitized surfaces in healthcare environments. Surfaces are recognized as being the most important reservoirs of bacteria and potential areas for the development of biofilms in healthcare facilities.^1^ Thus, this antimicrobial therapy becomes as an encouraging strategy to limit the risk of cross-contamination and nosocomial infections.^12,25^

Our group has recently reported the electrochemical formation of antimicrobial polymeric films bearing porphyrins as photoactive units and carbazol moieties as polymerization centers.^26,27^*In vitro* studies showed that these polymeric films were effective to photoinactivate microbes in planktonic media, and to eradicate biofilms. In addition, Comuzzi *et al.* reported the photokilling ability of polymeric materials obtained by imprinting a pentaphyrin derivative into electrodeposited dipyrromethane films.^28^ Although all these materials showed promising photokilling activity, the photostability of tetrapyrrolic macrocycle can be a disadvantage and a determining factor for practical applications. Taking this condition into account, fullerene C_60_ is a more photostable PS than porphyrinoid derivatives under visible light irradiation, since it undergoes relatively less photobleaching than tetrapyrrolic macrocycles.^29–32^ Blacha-Grzechnik *et al.* have recently demonstrated that films of polythiophene-C_60_ dyads, electrodeposited on indium–tin oxide (ITO) electrode, have the capability to produce O_2_(^1^Δ_g_) under light irradiation.^33^ Nyga *et al.* have proved that selenophene-fullerene polymeric dyads, electrochemically deposited, were also able to generate O_2_(^1^Δ_g_) after visible illumination.^34^

Within the group of electroactive molecules, 3,4-ethylenedioxythiophene (EDOT) is a versatile matrix to generate electrodeposited surfaces. EDOT can be readily electropolymerized and is a promising group for practical applications since several studies of electrodeposited films of poly(3,4-ethylenedioxythiophene) (PEDOT) composite materials have shown excellent stability and antifouling/antimicrobial properties.^35–38^ Thus, the derivatization of EDOT with a PS is an attractive idea to generate novel antimicrobial surfaces.

**Scheme 1:**
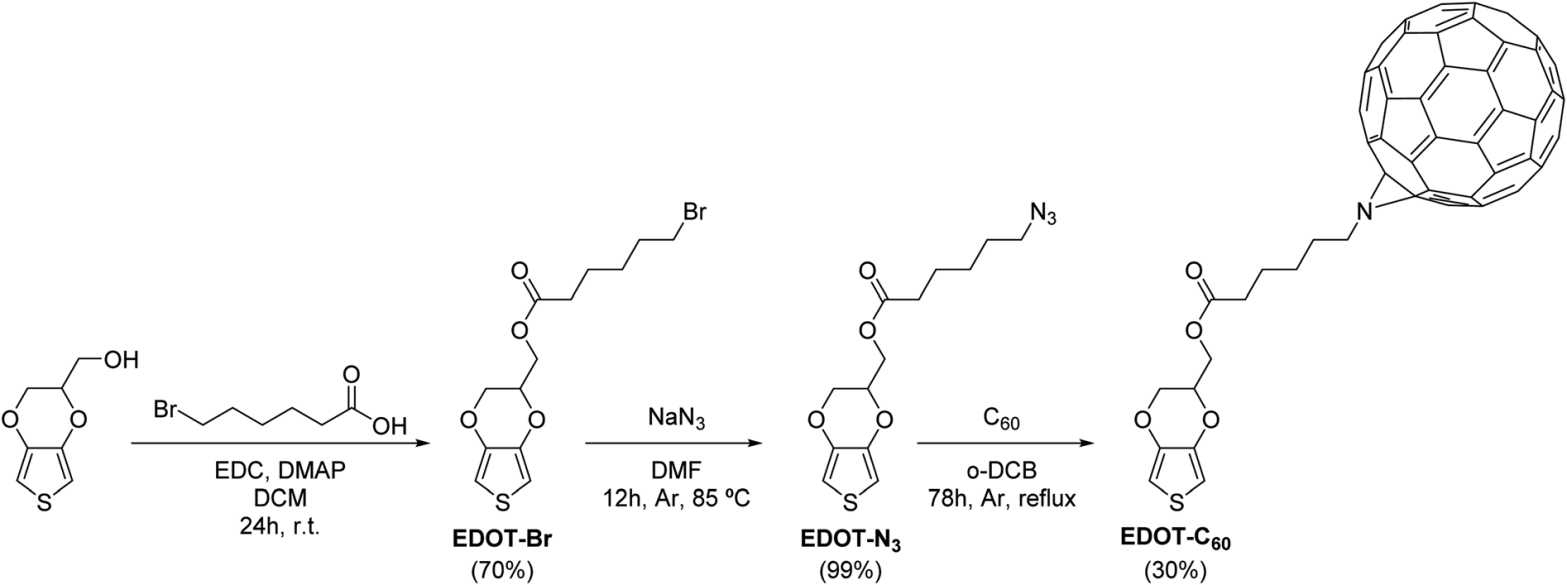
Synthetic pathway toward EDOT-C_60_.

In this work we constructed a dual functional material, which combines the photodynamic and photostable properties of fullerene C_60_ with the well-known polymerizable EDOT group. Our approach was based on the design and synthesis of an electroactive and photodynamic monomer (EDOT-C_60_), which contains an EDOT center covalently linked to a fullerene C_60_ moiety (Scheme 1). The electropolymerization process of EDOT-C_60_ led to the synthesis and deposition of polymeric films (PEDOT-C_60_), which were characterized by cyclic voltammetry and scanning electron microscopy (SEM). Their spectroscopic features and photodynamic properties were also studied and compared with fullerene C_60_. Photodynamic inactivation mediated by PEDOT-C_60_ films, were tested *in vitro* against *S. aureus*, a major colonizer of hospital environments. The antimicrobial efficiency of the photodynamic material was assessed inactivating pathogens in: planktonic suspension, adhered cells to photosensitizing surface and biofilms. Our outcomes demonstrate the development of a new photostable and photodynamic antimicrobial surface. These findings pave the way for the fabrication of new, easy to produce, fullerene-based antimicrobial surfaces.

## 2. Experimental sections

### 2.1 Synthesis

#### 2.1.1 EDOT-Br

4-(Dimethylamino)pyridine (DMAP; 54 mg, 0.46 mmol) and *N*-(3-dimethylaminopropyl)-*N*′-ethylcarbodiimide hydrochloride (EDC; 278 mg, 1.45 mmol) were successively added to a stirred solution of hydroxymethyl EDOT (EDOT-OH; 200 mg, 1.16 mmol) and 6-bromohexanoic acid (226 mg, 1.16) in anhydrous dichloromethane (DCM; 4 mL). The mixture was stirred at room temperature for 24 h under argon atmosphere. Then, DCM (20 mL) was added and the reaction mixture was washed with water (2 × 20 mL). The organic phase was dried with anhydrous MgSO_4_ and the solvent was removed under reduced pressure. The oily residue was purified by flash column chromatography (silica gel; DCM/cyclohexane, 1 : 1 → 1 : 0) to afford EDOT-Br (285 mg, 70%) as a yellow pale oil. ^1^H NMR (300 MHz, CDCl_3_) *δ* 6.36 (d, *J* = 3.6 Hz, 1H), 6.34 (d, *J* = 3.6 Hz, 1H), 4.42–4.34 (m, 1H), 4.33–4.30 (m, 2H), 4.25–4.01 (m, 2H), 3.40 (t, *J* = 6.7 Hz, 2H), 2.38 (t, *J* = 7.4 Hz, 2H), 1.88 (q, *J* = 7.1 Hz, 2H), 1.67 (q, *J* = 7.4 Hz, 2H), 1.53–1.46 (m, 2H). ^13^C NMR (75 MHz, CDCl_3_) *δ* 172.9, 141.2, 141.0, 100.0, 99.9, 71.4, 65.6, 62.2, 33.7, 33.4, 32.3, 27.6, 23.9. IR (film, *ν̃*): 762, 1024, 1119, 1184, 1252, 1377, 1427, 1454, 1485, 1732, 2868, and 2932 cm^−1^. MS *m*/*z* 348 (M^+^) (348.0031 calculated for C_13_H_17_BrO_4_S).

#### 2.1.2 EDOT-N_3_

EDOT-Br (170 mg, 0.49 mmol) and NaN_3_ (475 mg, 7.30 mmol) were dissolved in *N*,*N*-dimethylformamide (DMF; 7 mL) and stirred at 90 °C overnight under argon atmosphere. Then, DCM (25 mL) and water (25 mL) were added. The organic layer was extracted and washed with water (25 mL × 4). The organic solvent was removed under reduced pressure to afford EDOT-N_3_ (152 mg, 99%), as a yellow pale oil. ^1^H NMR (300 MHz, CDCl_3_) *δ* 6.36 (d, *J* = 3.5 Hz, 1H), 6.34 (d, *J* = 3.5 Hz, 1H), 4.42–4.33 (m, 1H), 4.33–4.29 (m, 2H), 4.25–4.20 (m, 2H), 3.27 (t, *J* = 6.8 Hz, 2H), 2.38 (t, *J* = 7.4 Hz, 2H), 1.76–1.55 (m, 4H), 1.49–1.38 (m, 2H). ^13^C NMR (75 MHz, CDCl_3_) *δ* 172.9, 141.2, 141.0, 100.0, 99.9, 71.4, 65.6, 62.2, 51.2, 33.7, 28.5, 26.2, 24.3. IR (film, *ν̃*): 1026, 1094, 1185, 1487, 1738, 2097, 2868 and 2934 cm^−1^. MS *m*/*z* 311 (M^+^) (311.0940 calculated for C_13_H_17_N_3_O_4_S).

#### 2.1.3 EDOT-C_60_

Fullerene C_60_ (463 mg, 0.64 mmol) and EDOT-N_3_ (100 mg, 0.32 mmol) were dissolved in dry 1,2-dichlorobenzene (*o*-DCB, 25 mL). The solution was refluxed for 36 h under argon atmosphere. Then, the organic solvent was removed under reduced pressure and the black solid residue was chromatographed (silica gel; toluene/cyclohexane, 1 : 1 → 1 : 0) to give EDOT-C_60_ (97 mg, 30%) as a dark brown powder. ^1^H NMR (300 MHz, CDCl_3_) *δ* 6.38 (d, *J* = 3.6 Hz, 1H), 6.35 (d, *J* = 3.6 Hz, 1H), 4.44–4.37 (m, 1H), 4.36–4.33 (m, 1H), 4.29–4.02 (m, 2H), 3.81 (t, *J* = 7.1 Hz, 2H), 2.50 (t, *J* = 7.3 Hz, 2H), 2.12–1.97 (m, 2H), 1.92–1.68 (m, 4H). ^13^C NMR (75 MHz, CDCl_3_) *δ* 173.2, 147.8, 146.8, 145.0, 144.7, 144.6, 144.5, 144.3, 144.2, 143.8, 143.7, 143.4, 143.1, 142.9, 142.8, 142.7, 142.6, 141.4, 141.2, 141.1, 140.8, 139.2, 138.5, 138.1, 137.3, 137.2, 136.2, 135.8, 133.7, 100.1, 100.0, 71.5, 65.7, 62.3, 51.4, 34.0, 29.2, 26.8, 24.7. IR (KBr, *ν̃*): 525, 571, 754, 1025, 1181, 1370, 1427, 1484, 1738, 2857 and 2931 cm^−1^. ESI-MS *m*/*z* 1005.1041 (M + H)^+^ (calculated for C_73_H_18_NO_4_S 1004.0957).

### 2.2 Polymer films electrodeposition

**Fig. 1:**
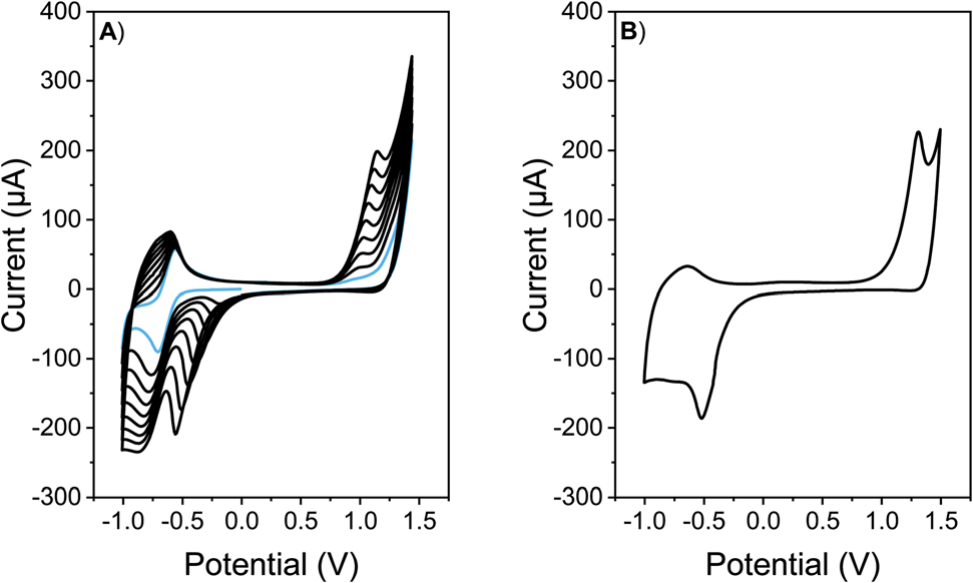
(A) Repetitive cyclic voltammograms of a 0.5 mM solution of EDOT-C_60_ in TBAPF_6_ (0.1 M)/DCM. Light blue line denotes the first cyclic voltammogram. (B) Cyclic voltammogram of a PEDOT-C_60_ film electro-deposited on ITO electrode in TBAPF_6_ (0.1 M)/DCM. Scan rate: 50 mV s^−1^.

The generation of polymeric films was performed by electrochemical methods, using a conventional three electrode glass cell. ITO electrodes (7 × 50 × 0.9 mm, Delta Technologies, Stillwater, MN, sheet resistance 8–12 Ω sq^−1^) were used as working electrodes, while a platinum mesh and a silver wire were used as counter electrode and pseudo-reference electrode, respectively. Applied potential was adjusted *versus* Saturated Calomel Electrode (SCE) formal potential using ferrocene as internal standard. DCM solutions containing monomer (0.5 mM) and tetrabutylammonium hexafluorophosphate (TBAPF_6_, 0.1 M) as supporting electrolyte were used for the electrodeposition. The monomer was polymerized and deposited over ITO surface by cyclic voltammetry (CV), cycling the electrode in the potential range between −1.0 and 1.4 V at 50 mV s^−1^ (Fig. 1).

### 2.3 Spectroscopic studies

UV-visible absorption measurements of EDOT-C_60_ were performed in air-equilibrated DCM solution at room temperature, using a quartz cell (10 mm path length). Absorption spectra of PEDOT-C_60_ films deposited on ITO were acquired by placing the sample in the spectrometer cell holder. The background correction was obtained by taking an UV-visible spectrum of an ITO electrode without the film.

### 2.4 Generation of O_2_(^1^Δ_g_)

The generation of O_2_(^1^Δ_g_) sensitized by PEDOT-C_60_ was determined by photooxidation of 9,10-dimethylanthracene (DMA) in DMF. This method was carried out in a quartz cell at room temperature. PEDOT-C_60_ surfaces were dipped in a solution of DMA (40 μM) and irradiated with light in a wavelength range between 455 and 800 nm (30 mW cm^−2^).^39^ A solution containing fullerene C_60_ and DMA (40 μM) was also irradiated (455–800 nm) and used as reference. The photooxidation rates of DMA were evaluated by following the decrease in the intensity of the DMA absorption band at *λ*_max_ = 379 nm. The observed rate constants *k*^DMA^_obs_ were determined from the slope of the plot ln(*A*_0_/*A*) *vs.* time. The quantum yield of O_2_(^1^Δ_g_) generation (*Φ*_Δ_) was calculated by direct comparison between the *k*^DMA^_obs_ of PEDOT-C_60_ and the reference (fullerene C_60_, *Φ*_Δ_ = 1).^40^

### 2.5 Detection of superoxide radical anion

The nitro blue tetrazolium (NBT) method was used to detect superoxide radical anion (O_2_˙^−^) formation in DMF.^41^PEDOT-C_60_ film was dipped in a solution containing 0.2 mM NBT and 0.5 mM reduced nicotinamide adenine dinucleotide (NADH) in 2 mL of DMF/water (9/1), and it was irradiated with visible light (90 mW cm^−2^) at different times under aerobic condition.^42^ The NBT reduction was followed by the increase of the absorbance at *λ* = 560 nm (diformazan formation). A fullerene C_60_ solution was also used for comparison. Control experiments were performed in absence of NBT, NAHD or PEDOT-C_60_.

### 2.6 Photooxidation of l-tryptophan

PEDOT-C_60_ film was dipped in a solution of 25 μM l-tryptophan (Trp) in DMF (2 mL) and irradiated with visible light (90 mW cm^−2^) under aerobic conditions. A fullerene C_60_ solution was used for comparison. The kinetics of Trp photooxidation were monitored by following the decrease of the fluorescence intensity (*I*) at *λ* = 343 nm. Trp was excited at *λ*_exc_ = 290 nm. The observed rate constants (*k*^Trp^_obs_) were obtained as previously reported.^43^

### 2.7 Photostability measurements

Photobleaching studies of PEDOT-C_60_ were carried out in phosphate-buffered saline (PBS).^26^PEDOT-C_60_ film was placed in a quartz cuvette containing PBS; then it was irradiated with visible light (90 mW cm^−2^) for 2 h under aerobic conditions. Absorption spectra were recorded at regular time intervals. The observed rate constant of photobleaching (*k*^P^_obs_) was determined as previously reported.^26^ The photobleaching lifetime (*τ*^P^_1/2_) was determined according to: *τ*^P^_1/2_ = ln(1/2)/*k*^P^_obs_.

### 2.8 Bacterial strains and preparation of cultures

The microorganism used was the strain of *S. aureus* ATCC 25923, which was previously characterized and identified.^44^ Microbial cells were grown aerobically in sterile condition overnight at 37 °C in 2 mL tryptic soy broth (TSB). An aliquot (60 μL) of the bacterial culture was aseptically transferred to 4 mL of fresh TSB and incubated at 37 °C to exponential phase of growth (absorbance 0.5 at 660 nm).

For the antibacterial drop-test, *S. aureus* in exponential phase of growth in TSB were centrifuged (3000 rpm for 15 min), and re-suspended in equal amount of PBS (pH = 7.4). Then, it was diluted to obtain ∼10^4^ colony forming unit (CFU) per milliliter. The additives sodium azide and mannitol were added from a stock solution in water (2 M and 1 M, respectively). Microorganism was incubated with sodium azide (50 mM) and mannitol (50 mM) for 30 min in dark at 37 °C before the photoinactivation experiment.

For the *S. aureus* biofilm formation on PEDOT-C_60_, 100 μL of the TSB culture was transferred to the 24-well microtiter plates containing 1 mL of PBS solution and 0.9 mL of TSB. PEDOT-C_60_ fragment (1 × 1 cm) was placed in each well and incubated for 24 h at 37 °C under continuous stirring. Then, the surface containing the biofilm were aseptically washed with PBS (2 mL × 2) in order to remove non-adherent cells. The back of the electrode (surface of glass without PEDOT-C_60_) was cleaned using a cotton swab with 70% v/v ethanol.

### 2.9 PDI experiments: planktonic cells and mechanism studies

The PDI was evaluated on *S. aureus* growing as planktonic cells. A drop (200 μL) of *S. aureus* suspension in PBS (∼10^4^ CFU mL^−1^) in the absence and in the presence of additives (sodium azide and mannitol) was deposited on the surface of the PEDOT-C_60_ (fragment of 1 × 1 cm) into a sterilized Petri dish. The PDI treatments in planktonic suspension were carried out with visible light irradiation (90 mW cm^−2^). PEDOT-C_60_ film with the drop was irradiated for 30 and 60 min. Then, viability of *S. aureus* was evaluated by serial dilution and plating on tryptic soy agar (TSA). Colonies formed were counted after 18 h of incubation at 37 °C in the dark.

### 2.10 PDI experiments: assembled biofilms

For the *S. aureus* biofilm inactivation, each PEDOT-C_60_ fragment containing the bacterial biofilm (24 h incubation) was washed with PBS (2 mL × 2) in order to remove non-adherent cells. The *S. aureus* biofilm (∼10^8^ CFU mL^−1^ cm^−2^) on PEDOT-C_60_ was irradiated with visible light under the same conditions described above. After PDI treatment, the surfaces were transferred to Pyrex brand culture tubes (13 × 100 mm) containing PBS (2 mL), and the biofilm was disrupted mechanically to take off the adhered bacteria. Cell suspensions were serially diluted 10-fold in PBS. From each dilution, 20 μL aliquots were streaked horizontally on TSA plates in sextuplicate.^45^ Viable microbial cells were monitored and the number of CFU was determined after ∼24 h incubation at 37 °C in the dark.

### 2.11 PDI of bacteria attached to the surface using fluorescence microscopy

Bacterial inactivation at the single cell level was performed using the methodology described by us and others but with some modifications.^46^ Two types of chambers capable of containing up to 100 μL of solution were ensembled. One of the chambers had a plastic cylinder glue on top of a previously coated PEDOT-C_60_ surface, while in the other (used as the control), the cylinder was glued on an ITO-coated surface. Bacteria were incubated in the homemade chambers for 20 min at 37 °C. After this period, the chamber was washed with successive cycles of PBS buffer, remaining only bacteria that were attached to the surface. Then, 95 μL of the buffer was added followed by addition of propidium iodide (PI) (5 μL) and incubated for 20 min. The final concentration of PI in the solution was 1 μM.

Fluorescence emission from the PI was monitored using an inverted fluorescence microscope, exciting with green light and employing a band pass filter (515/35). The power measured out of the objective was 0.9 mW cm^−2^. Fluorescence from the PI was filtered using an emission band pass filter (645/75). The inactivation experiments started with irradiation of the sample with visible light (3.1 mW cm^−2^) and the energy dose for all the experiments was 5.6 J cm^−2^.

A Carl Zeiss CP ACHROMAT 100× objective was used in all experiments. Fluorescence was collected through the same objective and was capture with a CMOS camera. Phase contrast images were also acquired for each experiment to colocalize cells in the imaging region.

### 2.12 Scanning electron microscopy of *S. aureus* biofilm on PEDOT-C_60_

*S. aureus* biofilm on PEDOT-C_60_ samples were prepared as previously reported.^47,48^ The samples were then covered with gold and examined with a scanning electron microscope. SEM images were obtained with a field emission scanning electron microscope FE-SEM, Sigma Zeiss (LAMARX facilities).

## 3. Results and discussion

### 3.1 Synthesis of EDOT-C_60_

The electroactive monomer EDOT-C_60_ was synthetized following the synthetic sequence shown in Scheme 1. First, commercially available hydroxymethyl-EDOT was esterified with 6-bromohexanoic acid in presence of EDC and DMAP in DCM to provide EDOT-Br in 70% yield. Instead of the commonly used DCC, EDC was used since the water-soluble EDC byproduct is easily removed during the aqueous work-up. EDOT-Br was then submitted to a nucleophilic substitution with sodium azide in DMF, cleanly furnishing the azide EDOT-N_3_ in essentially quantitative yield. Finally, a dipolar [1,3]-cycloaddition between EDOT-N_3_ and fullerene C_60_ was carried out by refluxing in anhydrous *o*-DCB. Under this condition, the desired cycloadduct EDOT-C_60_ was obtained in 30% yield. Thus, the synthetic sequence gave rise to EDOT-C_60_ in 21% overall yield after three straightforward and scalable steps.

The molecular structures of EDOT-C_60_ and its intermediaries were confirmed and determined by Fourier transform infrared (FT-IR) spectroscopy and nuclear magnetic resonance (NMR) (Fig. S1–S12†). FT-IR spectroscopy was used to quickly obtain diagnostic information about the synthetic transformations. The disappearance of the OH stretch from the EDOT-OH and the appearance of the carbonyl stretching band at 1740 cm^−1^ were distinctive signals for the first chemical transformation. In the second reaction step, the unique azide band at 2097 cm^−1^ was clearly visible in the IR spectrum.^49^ Besides, the success of the cycloaddition reaction was noticed by the disappearance of the azide absorption and the presence of the characteristic band of the organofullerene system at 525 cm^−1^.^50,51^

The ^1^H NMR spectra of the three EDOT derivatives exhibited akin features due to their structural similarity. The ^1^H NMR spectrum of EDOT-C_60_ displayed thiophene protons at *δ* = 6.38 and 6.35 ppm (*J* = 3.7 Hz) as a consequence of the asymmetric substitution of the EDOT moiety. Additionally, the methylene and methine protons of the dioxin ring gave rise to complex multiples between 4.0 and 4.5 ppm. Furthermore, the resonances of the methylenes of the alkyl side chain were observed between 1.70 and 3.90 ppm, whilst the incorporation of fullerene C_60_ was evidenced by deshielded proton signals, generated by the electron-withdrawing properties of fullerene C_60_.

In relation to the ^13^C NMR spectrum, EDOT-C_60_ combined the characteristic resonances of the EDOT residue and the alkyl side chain, together with the diagnostic resonances corresponding to the fullerene C_60_ moiety. It is well-known that the alkyl-azide cycloaddition to fullerene C_60_ can afford two aza-bridged regioisomers (Fig. S13†): a major product corresponding to the [5,6]-open dihydrofullerene and a minor one, corresponding to the [6,6]-closed structure.^52,53^ Interestingly, however, the [5,6]-open structure was selectively formed in EDOT-C_60_. This was determined from the carbon resonances corresponding to the fullerene C_60_ moiety, which were found in the range of 130–150 ppm, a spectral region attributable to sp^2^-type carbons.^54^ Further, the absence of signals corresponding to sp^3^ hybridized carbon atoms within the C_60_ framework (*δ* = 70–90 ppm) discarded the formation of a closed [6,6]-type structure.

### 3.2 Electrochemical formation of antimicrobial surface

Antimicrobial surfaces were generated by electrochemical polymerization and film deposition. This suitable and versatile methodology allows the polymer synthesis and film formation in one step at room temperature, avoiding solubility and/or stability requirements, that are necessary for polymer film formation by spin coating or vapor deposition methodologies.^26,55,56^ In order to explore the adequate conditions for electrochemical polymerization and film formation, CV analysis was conducted. Firstly, the redox behavior of EDOT-C_60_ monomer (0.5 mM) was investigated in an electrolyte solution containing TBAPF_6_ (0.1 M)/DCM over ITO electrode (Fig. 1A). The first cathodic scan of the monomer EDOT-C_60_ shows one reversible peak at −0.64 V. This peak was assigned to the first reduction of C_60_ moiety (Fig. 1A, light blue line).^57^ On the other hand, when a positive potential was applied, an irreversible oxidation process was detected at around 1.3 V, which can be ascribed to the formation of EDOT radical cations.^57^ These results are in agreement with the structural properties of EDOT-C_60_ monomer and prove that the main electronic characteristics of both moieties were retained in this new molecule. Furthermore, EDOT radical cations can react to form a PEDOT polymer.^58^ In fact, continuous cycling between −1.0 and 1.4 V produces increments in the oxidation–reduction currents after each new cycle, demonstrating the formation of an electrochemical active polymer on the electrode surface.

When the polymeric films onto electrodes are put into a DCM solution containing only supporting electrolyte, the redox response presents the oxidation/reduction peak currents corresponding to PEDOT and fullerene C_60_, respectively (Fig. 1B). A widening of the voltammetric peaks in the cathodic response is observed. This is common for organic polymeric films, due to the interaction between the redox centers in the solid state, changes in the solvation and charges diffusion phenomena.^59^ Moreover, when the number of cycles in the electrochemical deposition were modified, films with different absorption were produced (Fig. S14†).

**Fig. 2:**
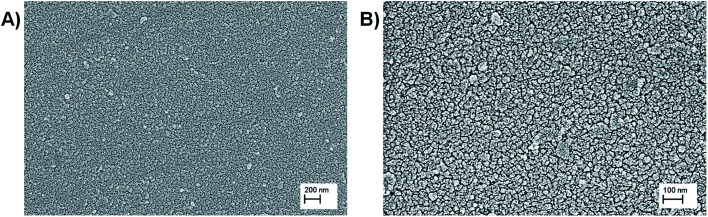
SEM imagines of PEDOT-C_60_ polymer film at different magnifications: (a) 23 000×, and (b) 57 000×.

The electrodeposited films were stable, with excellent adhesion properties to the electrode. The homogeneous distribution of the film on the surface was observed by optical microscopy and the obtained images are shown in Fig. S15.† Further, the surface morphology of the films was studied by SEM. As is shown in Fig. 2A, the SEM imagines of PEDOT-C_60_ demonstrated a total covering of the ITO electrode with the absence of pinholes or cracks, showing a smooth and homogeneous coating. At a higher magnification (Fig. 2B), a uniform and granulated surface formed by a compact and fused agglomeration was observed.

The synthetic design of the C_60_-containing monomer allowed the direct growth of a PEDOT backbone holding fullerene C_60_ pendant groups. This led to the formation of a polymeric film with a high fullerene content, corresponding to one fullerene C_60_ unit per polymer repeat unit.

### 3.3 Spectroscopic characterization

**Fig. 3:**
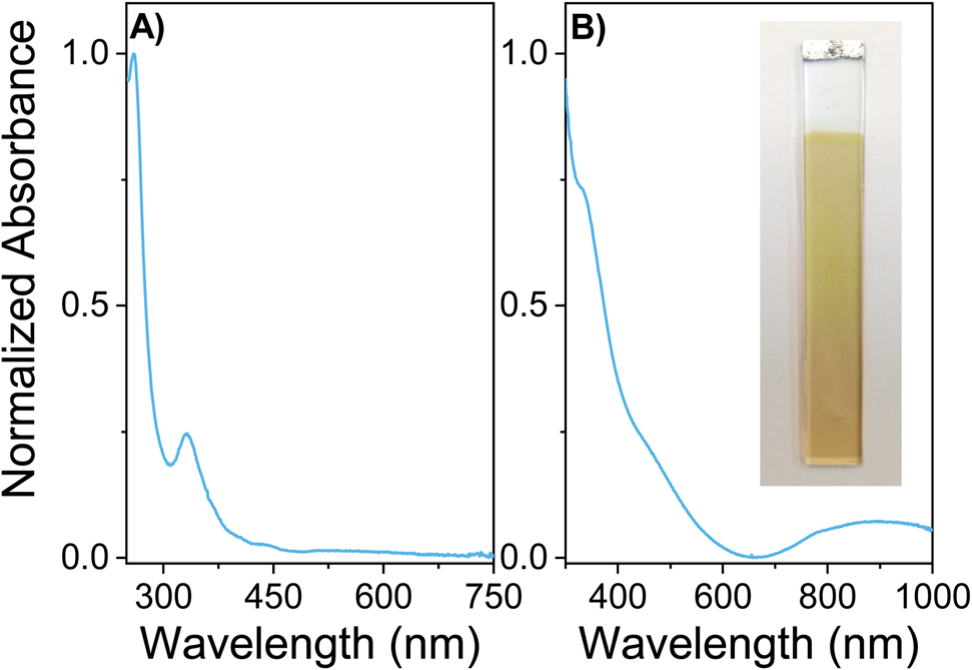
(A) Electronic absorption spectrum of EDOT-C_60_ monomer in DCM at room temperature. (B) Absorption spectrum of PEDOT-C_60_ film on ITO. The inset shows a photo of the polymer PEDOT-C_60_ deposited on ITO.

Spectroscopic properties of the monomer EDOT-C_60_ and the electrodeposited polymer PEDOT-C_60_ were studied in solution and thin solid films, respectively. Fig. 3 shows the absorption spectrum of EDOT-C_60_ in DCM solution as well as the spectrum of PEDOT-C_60_ deposited on ITO. Both spectra exhibited the typical absorption of fullerene derivatives, characterized by a strong absorption in the UV region and weaker absorptions in the visible region, with a tail extending to the near-IR region.

As it can be observed in Fig. 3A, EDOT-C_60_ shows the characteristic electronic transitions of azafulleroids with two main absorption bands at 260 and 330 nm, and a small band at 445 nm.^59,60^ Furthermore, the electronic transition between 420–440 nm, distinctive of [6,6] closed ring configuration,^60^ is not present in EDOT-C_60_ spectrum. This spectroscopic evidence also confirms the formation of an open [5,6]-aza-bridged adduct.

Fig. 3B shows the absorption spectrum of electrodeposited PEDOT-C_60_ film on ITO electrode. The inset in Fig. 3B allows to appreciate how the electropolymerization process of the monomer lead to the formation of a yellow-brownish coating. The PEDOT-C_60_ polymeric surface combines the spectroscopic features of the fullerene-based chromophore and the conjugated polymeric chain of PEDOT.^59,61^ The electronics transitions corresponding to the fullerene absorption are broadened and bathochromically shifted compared to the monomer bands in solution. This is a consequence of the extensively aggregated system formed in the polymeric matrix. The broad absorption band center at 900 nm could be attributed to the π–π* transition of the extended conjugated polymer chain of PEDOT and to the partial oxidation of the film.^61,62^ Besides, the spectrum of PEDOT-C_60_ confirms not only that the electropolymerization of the EDOT-C_60_ generates a polymeric surface containing fullerene C_60_, but also that the spectroscopic properties of the fullerene-based chromophore are retained, evidencing that the chromophore structure is not altered during the electrodeposition process.

### 3.4 Polymeric surface photostability

One of the main requisites for the development of photoactive antimicrobial coatings is the photostability of the PS attached to the surface. This plays a predominant role in the reuse of the surface, which is essential for practical applications. Photodegradation processes, caused by exposure to light, affect the antimicrobial efficacy. Consequently, photobleaching studies of the PEDOT-C_60_ polymeric coatings were carried out using visible light irradiation.

The film photostability was investigated in PBS by monitoring the decrease of the absorption at 369 nm. PEDOT-C_60_ surfaces were irradiated for 2 h and the absorption spectra were acquired at regular intervals of time. Fig. S16† shows the insignificant spectroscopic variations exhibited for PEDOT-C_60_, with a negligible loss of absorbance after visible light exposure. Furthermore, discoloration of the surface was not observed. Thus, the polymeric surfaces can be exposed to visible irradiation retaining their spectroscopic characteristics.

**Fig. 4:**
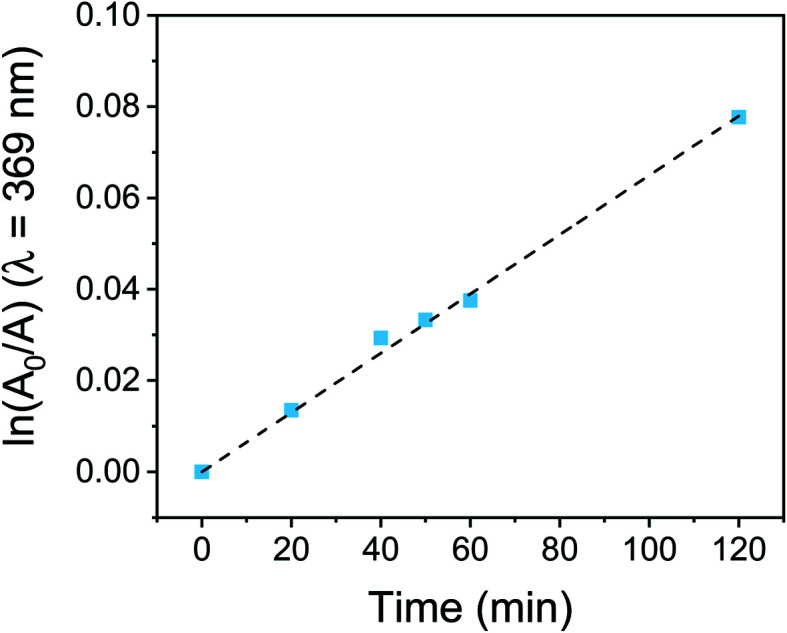
First-order plot for the photodegradation of PEDOT-C_60_ surface dipped in PBS and irradiated with visible light.

As it can be observed in Fig. 4, the slight photodegradation process followed a first-order kinetic with the irradiation time. The value of the *k*^P^_obs_, obtained from the slope of the previous plot, was 6.53 × 10^−4^ min^−1^. This value was used to calculate the photodegradation lifetime, obtaining a *τ*^P^_1/2_ of 17.7 h in PBS. Although it is not possible to compare photobleaching results obtained by other groups due to the different experimental conditions, our outcomes showed that PEDOT-C_60_ exhibited higher photostability (almost two times more) than our previously reported porphyrin-based antimicrobial surface under similar conditions.^26^ This demonstrates that PEDOT-C_60_ films exhibit a high photostability, which gives them long lifetime with good performance for practical applications.

PEDOT-C_60_ photostability can be explained considering that fullerene moieties are highly resistant to photobleaching.^63,64^ Most of the conventional PS are entirely decomposed, losing their photodynamic action at relatively low energy dose; while fullerene conserves its photoactivity after very high fluences.^63^ In addition, fullerene C_60_ moieties in PEDOT-C_60_ are embedded in the polymeric matrix, which could result in an opportune steric hindrance against the ROS that are responsible for the photooxidation reactions.

### 3.5 Photosensitized generation of O_2_(^1^Δ_g_)

After a detailed characterization of the PEDOT-C_60_ polymeric film, its potential as an antimicrobial surface was first studied by assessing its ability to produce O_2_(^1^Δ_g_) under light irradiation. To that end, decomposition of DMA photoinduced by PEDOT-C_60_ was used to monitor the O_2_(^1^Δ_g_) generation.^26,65^ In this method, DMA quenches O_2_(^1^Δ_g_) by a rapid chemical reaction, producing the endoperoxide derivative.^66^ The photodecomposition reaction was monitored by UV-visible spectroscopy following the decrease of the DMA absorption band at 379 nm.^26^

In order to evaluate the O_2_(^1^Δ_g_) production photosensitized by PEDOT-C_60_, the surface was dipped in an air-equilibrated solution of DMA in DMF and it was then irradiated with light (455–800 nm). Absorption spectra acquired at different irradiation times showed the gradual decrease of the absorption bands of DMA (Fig. S17†), indicating that O_2_(^1^Δ_g_) was produced by the photosensitizing action of PEDOT-C_60_ polymeric films. It is worth to mention that PEDOT-C_60_ films were not dissolved during the kinetic study, which was evidenced by the absence of their absorption bands in the acquired spectra.

**Fig. 5:**
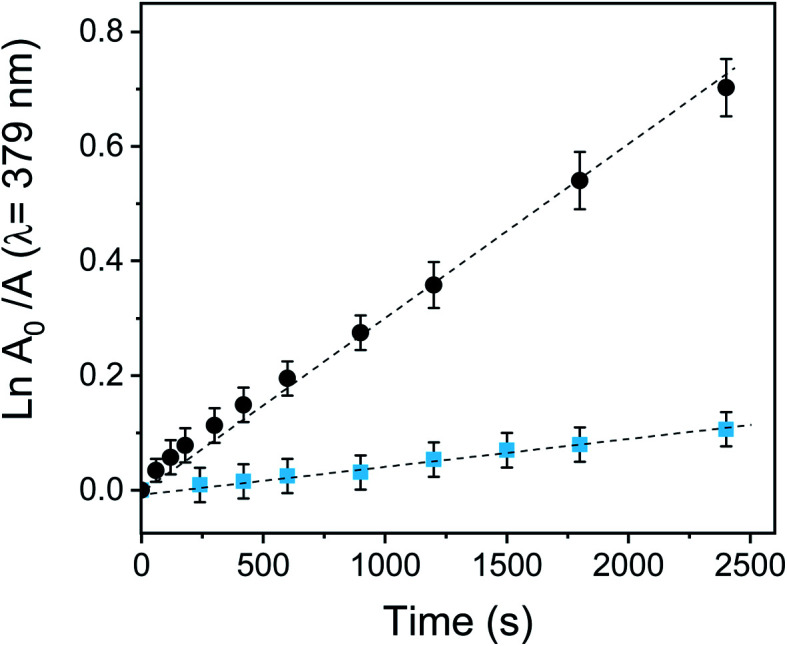
First-order kinetic data fitting for the photooxidation of DMA in DMF sensitized by PEDOT-C_60_ (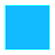) and fullerene C_60_ (●). *λ*_irr_ = 455–800 nm. Values represent mean ± standard deviation (error bars) of three consecutive experiments.

The consumption of DMA was also compared with a solution of fullerene C_60_ in DMF. The spectroscopy decay of DMA produced by the reference at different irradiation times is shown in Fig. S17.† As is shown in Fig. 5, DMA photooxidation exhibited a pseudo first-order kinetic for both systems but with different rates of O_2_(^1^Δ_g_) production. The values of *k*^DMA^_obs_ were determined from the slopes of these plots and are gathered in Table 1.

**Table 1:** Photodynamic properties of PEDOT-C_60_ and the reference fullerene C_60_ in DMF

	*k* ^DMA^_obs_ a (s^−1^)	*Φ*_Δ_	*k* ^Trp^_obs_ a (s^−1^)
PEDOT-C_60_	(4.55 ± 0.02) × 10^−5^	0.16 ± 0.02	(8.78 ± 0.06) × 10^−5^
Fullerene C_60_	(2.87 ± 0.02) × 10^−4^	1^b^	(1.12 ± 0.10) × 10^−4^

a Fitting error reported.

b Value from ref. 40.

The quantum yield of O_2_(^1^Δ_g_) production (*Φ*_Δ_) was calculated by comparing the *k*^DMA^_obs_ value for the electrodeposited film with that for the reference (fullerene, *Φ*_Δ_ = 1).^40^ Despite fullerene C_60_ has an efficient photodynamic activity with a high O_2_(^1^Δ_g_) quantum yield production, the *Φ*_Δ_ value for PEDOT-C_60_ was 0.16. This yield is appropriate since O_2_(^1^Δ_g_) generation takes place on the surface of PEDOT-C_60_. Also, the production efficiency of O_2_(^1^Δ_g_) of the film is lower since the aggregation processes in the solid state could be reducing the *Φ*_Δ_.^32,67^

The obtained *Φ*_Δ_ value not only confirms that PEDOT-C_60_ is able to generate O_2_(^1^Δ_g_), but also that it is more efficient compared to previous electrodeposited polymers.^26,27,68^ Furthermore, the O_2_(^1^Δ_g_) production demonstrates that a photodynamic mechanism type II occurs when PEDOT-C_60_ is irradiated with light.

### 3.6 Generation of superoxide radical anion

The capability of PEDOT-C_60_ films to photosensitized ROS thorough a type I mechanism was studied by following the production of superoxide radical anion (O_2_˙^−^). The NBT method was used to determine the generation of this ROS. NBT is a trapping reagent that is reduced by O_2_˙^−^ to form diformazan. This photosensitized reduction is favored by the presence of reducing agents such as NADH and polar solvents.^69,70^ NADH-mediated O_2_˙^−^ formation was monitored spectroscopically by following the increase of diformazan absorption band at *λ* = 560 nm (Fig. S18†).

**Fig. 6:**
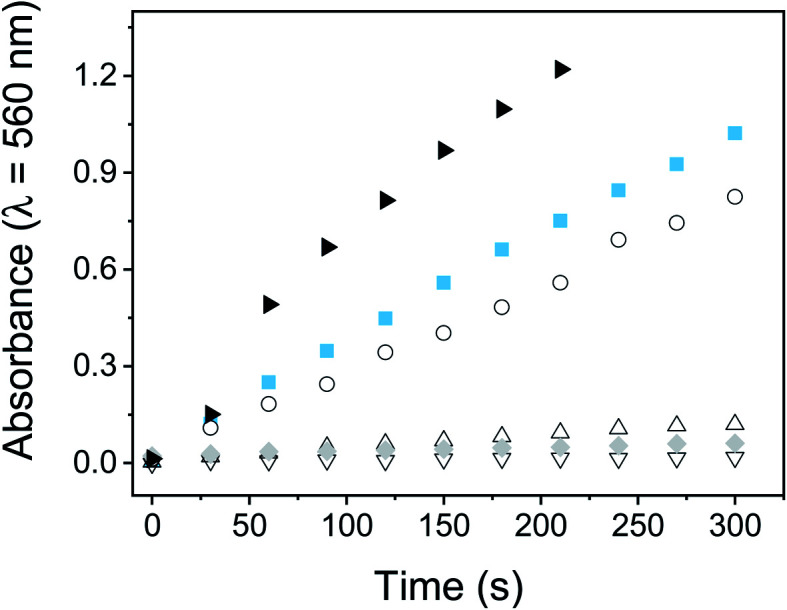
O_2_˙^−^ generation monitored by the NBT method following the increase in the absorption at 560 nm. Samples contain: PEDOT-C_60_, NBT and NADH (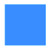); fullerene C_60_ solution, NBT and NADH (▶); NBT and NADH (○); PEDOT-C_60_ and NTB (△); PEDOT-C_60_ and NADH (▽); PEDOT-C_60_, NBT and NADH, dark control (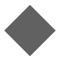). [NBT] = 0.2 mM and [NADH] = 0.5 mM.

Fig. 6 shows the increase of diformazan absorption band as a function of the irradiation time when PEDOT-C_60_ and fullerene C_60_ were irradiated with visible light in air-equilibrated DMF. As it can be observed, the reduction of NBT was not observed for control experiments: absence of NADH or NBT, and PEDOT-C_60_ in dark. On the other hand, although both PEDOT-C_60_ and fullerene C_60_ showed higher absorption than NADH + NBT control, fullerene C_60_ exhibited a higher production of diformazan. Again, this can be explained because O_2_˙^−^ is produced in the interface between the film and the solution.

These results confirm that a type I mechanism takes place when the polymeric films are irradiated. Up to this point, O_2_(^1^Δ_g_) and O_2_˙^−^ are produced by PEDOT-C_60_ films after light irradiation. This is in concordance with the capability of the fullerene C_60_ to generate ROS through both type I and II pathways.^27,30,43^

### 3.7 Photooxidation of Trp

Photooxidation of Trp is a useful model to study the photodynamic properties, because it could be a target molecule of the photodynamic effect induced by PDI treatments. Trp is located as a residue on protein chains; therefore modifications in its chemical structure can produce significant alterations in the physicochemical properties of the proteins and subsequently lead to a cellular damage.^71^ Trp can be efficiently photooxidized by both type I and type II photomechanisms.^71^ It is one of the most susceptible amino acids to oxidation by radicals and O_2_(^1^Δ_g_), with significant rates under physiological conditions.^71^

**Fig. 7:**
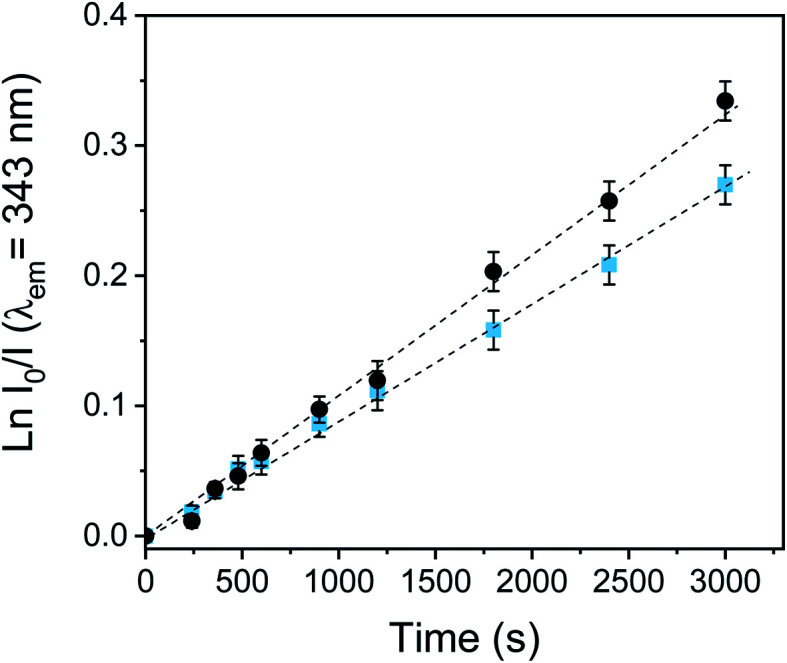
First-order plots for the oxidation of Trp (20 mM) photosensitized by the PEDOT-C_60_ surface (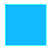) and fullerene C_60_ (●) in DMF solution, *λ*_irr_ = 455–800 nm.

The photooxidation of Trp sensitized by PEDOT-C_60_ films and fullerene C_60_ was carried out in air-equilibrated DMF, following the decrease of the Trp emission band at 343 nm (see Fig. S19†). As it can be observed in Fig. 7, the photodecomposition of Trp exhibited a pseudo-first order kinetic. The values of the *k*^Trp^_obs_ for Trp degradation were obtained from the slope of these plots (Table 1). The two studied systems photodecomposed Trp at similar rates. The *k*^Trp^_obs_ value for PEDOT-C_60_ was similar to that found for fullerene C_60_, with *k*^Trp^_obs_ values of 8.78 × 10^−5^ s^−1^ and 1.12 × 10^−4^ s^−1^ for PEDOT-C_60_ and fullerene C_60_, respectively. This result discloses that Trp is a key target of the photodynamic action prompted by the electrodeposited films.

Comparing the *k*_obs_ values for DMA and Trp, a high ration value of *k*^film^_obs_/*k*^fullerene^_obs_ was obtained for the Trp decomposition (*k*^film^_obs_/*k*^fullerene^_obs_ = 0.78) in comparation with the O_2_(^1^Δ_g_) production observed in DMA photooxidation (*k*^film^_obs_/*k*^fullerene^_obs_ = 0.16). Therefore, an electron transfer pathway could also be contributing to the Trp decomposition in DMF. PEDOT polymeric chain could absorb visible light, producing a photoinduced charge-separated state, as it was previously reported for a similar C_60_-containing electropolymer.^59^ Thus, fullerene C_60_ may be reduced to its radical anion (C_60_˙^−^) by electron transfer from the PEDOT, and consequently, C_60_˙^−^ could also transfer an electron to O_2_(^3^Σ^−^_g_) producing O_2_˙^−^.^72–74^

### 3.8 Photoinactivation of *S. aureus* cell suspension and mechanism studies

The inactivating capability of this photoactive material was investigated *in vitro* by depositing a drop of a *S. aureus* suspensions on the PEDOT-C_60_ surface. This antibacterial drop-test can serve to determine the antimicrobial action of the film to inactivate *S. aureus* cells in contaminated water. In fact, it has already been used to evaluate the capacity of electrogenerated polymeric films to inactivate microorganisms.^26,27^ In this approach, 200 μL of PBS containing ∼10^4^ cells was deposited on PEDOT-C_60_ and irradiated with visible light.

**Fig. 8:**
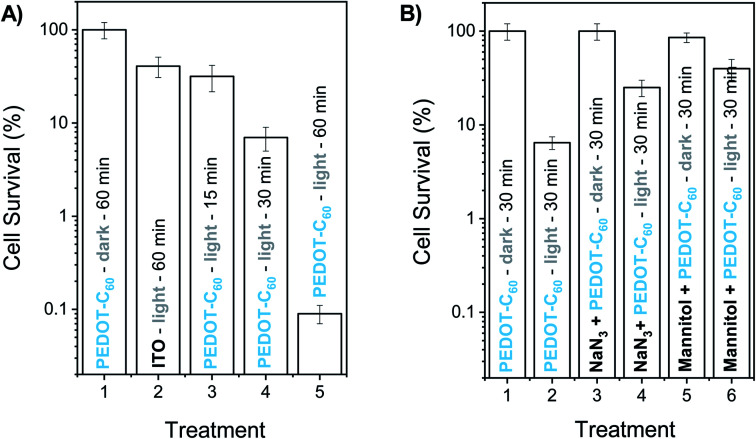
Survival of *S. aureus* (∼10^4^ CFU mL^−1^) cell suspensions in PBS depositing a drop (200 μL) with the cells on PEDOT-C_60_. (A) (1) Control: cells deposited on PEDOT-C_60_ and kept in dark 60 min. (2) Control: cells deposited on ITO and irradiated 60 min. (3) Cells deposited on PEDOT-C_60_ and irradiated 15 min. (4) Cells deposited on PEDOT-C_60_ and irradiated 30 min. (5) Cells deposited on PEDOT-C_60_ and irradiated 60 min. (B) (1) Control: cells deposited on PEDOT-C_60_ and kept in dark 30 min. (2) Cells deposited on PEDOT-C_60_ and irradiated 30 min. (3) Cells containing 50 mM sodium azide deposited on PEDOT-C_60_ and kept in dark 30 min. (4) Cells containing 50 mM sodium azide deposited on PEDOT-C_60_ and irradiated 30 min. (5) Cells containing 50 mM mannitol deposited on PEDOT-C_60_ and kept in dark 30 min. (6) Cells containing 50 mM mannitol deposited on PEDOT-C_60_ and irradiated 30 min. Samples were irradiated with visible light.

Fig. 8A shows the survival of bacterial cells after different irradiation times. Control experiments indicated that the viability of *S. aureus* was unaffected by keeping the cells on PEDOT-C_60_ surfaces in the dark for 60 min (Fig. 8A, treatment 1). In addition, after 60 min of irradiation, <1 log decrease was found for the ITO-coated glass (Fig. 8A, treatment 2). On the other hand, the viability of *S. aureus* cells deposited on PEDOT-C_60_ films was dependent on the light exposure time. A slight inactivation (∼1 log) was detected after 15 and 30 min of irradiation (Fig. 8A, treatment 3 and 4). While a ∼3.1 log decrease of *S. aureus* survival was produced after 60 min of irradiation (Fig. 8A, treatment 5). This result represents a value higher than 99,9% of cellular inactivation. Therefore, from all these data, it is inferred that the bacterial killing was produced by the photosensitization effect of the PEDOT-C_60_ polymeric film.

These findings can be compared to previous reports of PDI experiments carried out with carbon nanotubes (CNTs) which, together with fullerene C_60_, are the two most typical carbon-based nanomaterials. Although it is not correct to compare *in vitro* outcomes due to the diverse experimental conditions (number of cells, energy dose, PS concentration, *etc.*), our results showed that PEDOT-C_60_ presented a similar antimicrobial action than previously reported carbon-based nanomaterials. Serrano-Aroca *et al.* have shown that irradiation of carbon nanomaterials significantly improved their antibacterial properties, reaching a 99.7% inactivation of *S. aureus* after 3 h of LED irradiation.^75^ In addition, PEDOT-C_60_ presented a similar photoinactivation capability to porphyrin-CNT conjugates irradiated with visible light for 1 h.^76^

The effect of two scavengers of ROS (azide ion and mannitol) was also investigated in order to obtain information about the photodynamic mechanism involved in the *in vitro* photoinactivation. Sodium azide is a quencher of intracellular O_2_(^1^Δ_g_),^77^ while mannitol acts as a scavenger of O_2_˙^−^ and hydroxyl radical.^78^ According to this, 200 μL of PBS containing ∼10^4^ cells and 50 mM sodium azide was deposited on PEDOT-C_60_ surface. No toxicity was detected using this concentration of azide ion in the dark (Fig. 8B, treatment 3). After 30 min irradiation, a lower cell inactivation was obtained in presence of sodium azide (Fig. 8B, treatment 4) compared to the treatment without the ROS scavenger (Fig. 8B, treatment 2). The addition of azide ion quenched the O_2_(^1^Δ_g_), producing a protective effect on microbial cells. On the other hand, the presence of 50 mM mannitol was not toxic for bacteria cells on PEDOT-C_60_ in the dark (Fig. 8B, treatment 5). After 30 min of irradiation, the photokilling mediated by the surface exhibited a protective effect of around 1 log in suspensions containing mannitol (Fig. 8B, treatment 6), similar to the previous result. These outcomes indicate that two photooxidative pathways of action can take place under *in vitro* conditions, which is in concordance with the photodynamic studies carried out in solution.

### 3.9 Photoinactivation of *S. aureus* cells anchored to the surface

A common way bacterial infections spread is by surface contact transmission. This occurs particularly in common uses areas and facilities such as lobbies, doors, restrooms, dinners, and even public transport. Persons can also become infected by touching a contaminated surface and then touching their eyes, nose, mouth, and ears before washing hands. Another way of getting a disease transmitted by pathogens is by direct contact with an open wound, and the most common infection-causing bacteria is *S. aureus*.^79^ In this context, we have tested the inactivation capability of the surface monitoring bacterial inactivation at the single-cell level. This methodology was previously developed by us and others,^46^ and offers a simple and effective way of observing inactivation on a transparent surface employing PI as a cell death marker. Interaction of the dye with DNA or RNA duplexes emits red fluorescence after permeation through a ROS disrupted membrane on the surface of the material.^80^ This experiment may be considered as a proof of concept of airborne aerosolized contaminated water on a surface.

**Fig. 9:**
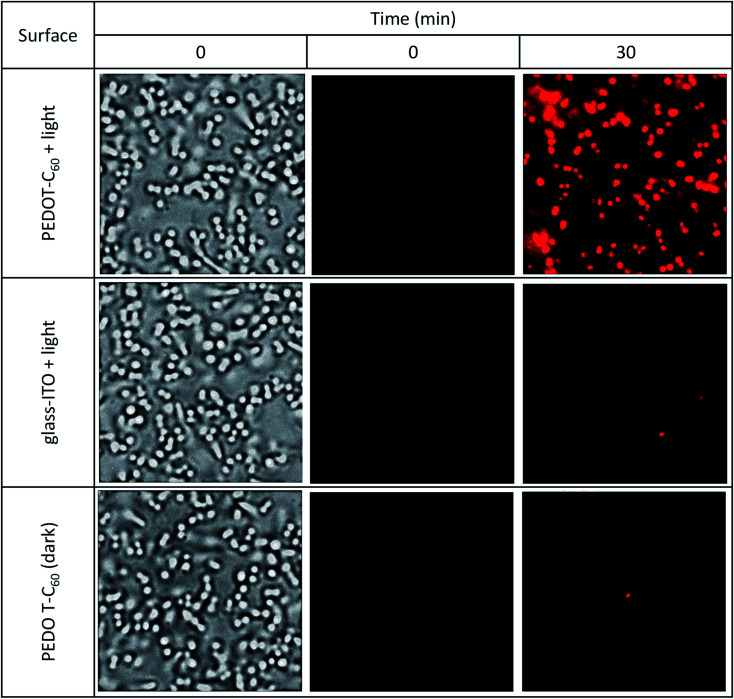
Bright-field (first column) and fluorescence microscopy (second and third columns) images of *S. aureus* on the surface of a coverslip coated with PEDOT-C_60_ + light and the controls, ITO with no coating + light and a PEDOT-C_60_ surface in the dark. [PI] = 1 μM. Light dose: 5.6 J cm^−2^.

As it can be observed in Fig. 9 top panel, a complete inactivation of the pathogens was achieved by PEDOT-C_60_ photoactive material after applying the therapy for 30 min and a low energy dose (5.6 J cm^−2^). Control experiments were done using a glass coated ITO electrode lacking the polymer but treated with the same energy dose, and a coated PEDOT-C_60_ coverslip in dark conditions (Fig. 9, middle and bottom panels). These experiments show no cell death as was expected. In a previous work, electrodepositing a biscarbazol-dendrimeric Zn(ii) porphyrin monomer, bacteria death was observed after applying the therapy for 7.5 min (0.3 J cm^−2^). The almost 20 times difference observed between these two surfaces can be attributed to the low absorption of visible light by the fullerene. However, the combination EDOT-fullerene offers a better control over the electrodeposition process and a higher photostability in comparison with the tetrapyrrolic macrocycle. In addition, the most relevant difference is that EDOT-C_60_ monomer requires only 3 simple synthetic steps, while biscarbazol-dendrimeric Zn(ii) porphyrin monomer required a more complex sequence of ten steps to prepare the PS substituted with the electropolymerizable groups.^26^

### 3.10 Photoinactivation of *S. aureus* biofilm

The antibacterial activity photoinduced by the PEDOT-C_60_ was also evaluated against *S. aureus* biofilm. This clinically important bacteria capable of growing on medical instruments and natural and inert surfaces is one of the main causes of the propagation of antibiotic resistance and the transmission of nosocomial infections.^7,8^ The biofilm physiology enables bacteria to survive prolonged exposure to antibiotics, in comparison with the cells in planktonic media.^81^

**Fig. 10:**
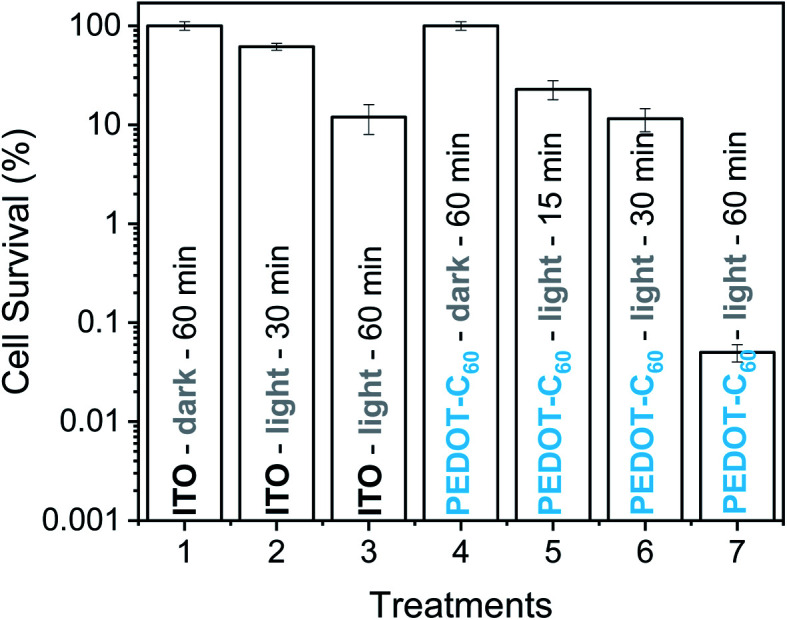
Survival of *S. aureus* biofilm on ITO electrode (treatments 1–3) and on PEDOT-C_60_ (treatments 4–7). (1) Control: biofilm on ITO electrode kept in dark 60 min. (2) Control: biofilm on ITO electrode irradiated 30 min. (3) Control: biofilm on ITO electrode irradiated 60 min. (4) Control: biofilm on PEDOT-C_60_ kept in dark 60 min. (5) Biofilm on PEDOT-C_60_ irradiated 15 min. (6) Biofilm on PEDOT-C_60_ irradiated 30 min. (7) Biofilm on PEDOT-C_60_ irradiated 60 min. Samples were irradiated with visible light.

To study the capability of PEDOT-C_60_ to photoinactivate *S. aureus* biofilms, the surface was immersed into a *S. aureus* suspension and it was incubated for 24 h in order to form the biofilm. Then, it was submitted to different irradiation times (15, 30 and 60 min) with visible light. Survival of *S. aureus* biofilms formed on the PEDOT-C_60_ is shown in Fig. 10. Control experiments confirmed that the viability of the bacteria was unaffected by dark incubation on ITO electrode and PEDOT-C_60_ (Fig. 10, treatments 1 and 4). In addition, a slight inactivation lower than 1 log was found after 30 and 60 min of irradiation of the biofilm on ITO (Fig. 10, treatment 2 and 3). When the biofilms on PEDOT-C_60_ were irradiated for 15 or 30 min, a small and similar photosensitizing activity was found (Fig. 10, treatments 5 and 6). However, PEDOT-C_60_ showed a higher photosensitizing activity after 60 min of irradiation (Fig. 10, treatment 7), producing a >3 log decrease of biofilm survival, which represent >99.9% of cell photoinactivation. All these outcomes reveal that the biofilm eradication was produced by the photosensitizing action of PEDOT-C_60_. Hence, the combination of the electrodeposited polymeric films and visible light makes them promising self-sterilizing surfaces with a high antimicrobial action.

**Fig. 11:**
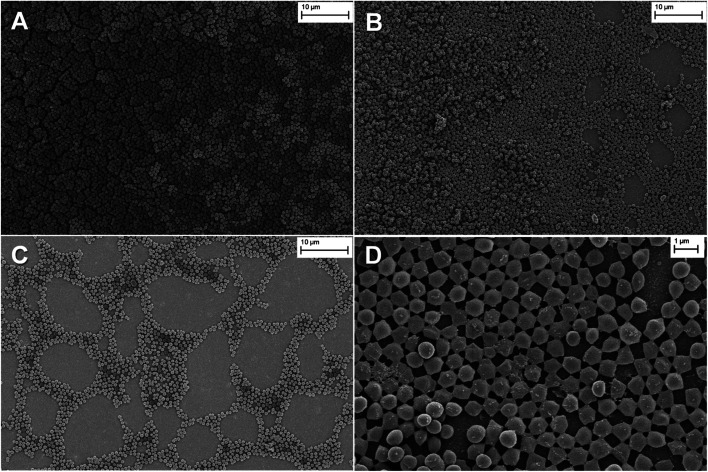
SEM images of *S. aureus* biofilm on PEDOT-C_60_. (A) Control: *S. aureus* biofilm on PEDOT-C_60_ keeping in the dark; (B) *S. aureus* biofilm on PEDOT-C_60_ exposed to visible light for 30 min; (C) *S. aureus* biofilm on PEDOT-C_60_ exposed to visible light for 60 min; (D) *S. aureus* biofilm on PEDOT-C_60_ exposed to visible light for 60 min at a higher magnification.

The morphology and surface characteristics of *S. aureus* biofilm on PEDOT-C_60_ were analyzed by SEM. Large cellular aggregates were observed for biofilms on PEDOT-C_60_ kept in the dark (Fig. 11A). Different irradiation times with visible light cause a time-dependent disruption of the biofilms: after 30 min irradiation a significant reduction in the number of adhered bacteria and size of the aggregates were observed (Fig. 11B); while biofilms on PEDOT-C_60_ exposed to 60 min of irradiation turned into small clusters and a pronounced alteration of biofilm morphology (Fig. 11C). The effect can be more clearly evidenced in the magnified image showed in Fig. 11D. Thus, the antibacterial activity and the efficiency of the PDI treatment induce by PEDOT-C_60_ films could also be confirmed by SEM micrographs.

## 4. Conclusions

A suitable and straightforward method to produce antimicrobial and self-sterilizing surfaces based on a polymerizable fullerene derivative was established. This approach involved the design and synthesis of an electroactive monomer, which contains an electropolymerizable center of EDOT linked to a fullerene moiety. The monomer EDOT-C_60_ was easily obtained after three simple synthetic steps. The decoration of the heterocyclic EDOT unit with fullerene C_60_ substituent ensured the facile electropolymerization process, leading to the formation of polymeric films with pedant fullerene units. Cyclic voltammetry and UV-visible spectroscopy studies showed that fullerene units were not altered during the electropolymerization process, conserving the main photophysical characteristics of the chromophore in the polymer matrix. Fullerene C_60_ pendant groups in PEDOT chain also retain their photodynamic properties, being able to generate ROS through both type I and type II mechanisms. The PEDOT-C_60_ electrodeposited film exhibited good photostability thanks to the low photodegradation of fullerene C_60_. Thereby, in this work we achieve a good combination of photostability and high photodynamic activity reached by the presence of fullerene C_60_ in the polymeric chain. Hence, this photoactive material would result useful for practical applications.

The antimicrobial capability of the surfaces was evaluated in three different situations that can contaminate a surface: planktonic cells, bacteria attached to the surface and biofilms. In all cases, the PDI treatment showed an inactivation higher than 99.9%. Thus, our approach can be considered bactericidal or antimicrobial according to the American Society of Microbiology,^82^ since a decrease higher than 3 log CFU (photoinactivation >99.9%) was achieved for the three different conditions. Our outcomes demonstrated that this highly efficient photoactive self-sterilizing coating could make an important contribution to control the microbial proliferation and to maintain aseptic conditions, reducing the infections caused by surface-adhering pathogens.

## Conflicts of interest

There are no conflicts to declare.

## Supplementary Material

RA-011-D1RA03417K-s001
