# Supplementary material for: Photoactive antimicrobial coating based on a PEDOT-fullerene C60 polymeric dyad
Source: RSC Adv. 2021 Jul 5;11(38):23519–32. doi: 10.1039/d1ra03417k (PMC9036534; doi:10.1039/d1ra03417k)
Supplement: RA-011-D1RA03417K-s001 [file RA-011-D1RA03417K-s001.pdf]

Electronic Supplementary Material (ESI) for RSC Advances.
This journal is © The Royal Society of Chemistry 2021

Photoactive Antimicrobial Coating Based on a PEDOT-Fullerene C_{60} Polymeric Dyad

Eugenia Reynoso[1], Andrés M. Durantini[1], Claudia A. Solis[2], Lorena P. Macor[2], Luis A. Otero[2], Miguel A. Gervaldo[2], Edgardo N. Durantini[1] and Daniel A. Heredia[1]*.

1 - IDAS-CONICET, Departamento de Química, Facultad de Ciencias Exactas Físico-Químicas y Naturales, Universidad Nacional de Río Cuarto, Agencia Postal Nro. 3, X5804BYA Río Cuarto, Córdoba, Argentina.

2 - IITEMA-CONICET, Departamento de Química, Facultad de Ciencias Exactas Físico-Químicas y Naturales, Universidad Nacional de Río Cuarto, Agencia Postal Nro. 3, X5804BYA Río Cuarto, Córdoba, Argentina.

Table of Contents

Description	Page Nº
**Experimental Procedures: General, Controls and Statistical Analysis**	S-3
**Figure S1**. ^{1}H and ^{13}C NMR spectra of **EDOT-Br**	S-6
**Figure S2**. HSQC and HMBC NMR spectra of compound **EDOT-Br**	S-7
**Figure S3**. COSY NMR spectrum of compound **EDOT-Br**	S-8
**Figure S4**. 1H and 13C NMR spectra of compound **EDOT-N_3**	S-9
**Figure S5**. HSQC and HMBC NMR spectra of compound **EDOT-N_3**	S-10
**Figure S6**. COSY NMR spectrum of compound **EDOT-N_3**	S-11
**Figure S7**. ^{1}H and ^{13}C NMR spectra of compound **EDOT-C_{60}**	S-12
**Figure S8**. HSQC and HMBC NMR spectra of compound **EDOT-C_{60}**	S-13
**Figure S9**. COSY NMR spectrum of compound **EDOT-C_{60}**	S-14
**Figure S10**. IR spectrum of compound **EDOT-Br**	S-15
**Figure S11**. IR spectrum of compound **EDOT-N_3**	S-15
**Figure S12**. IR spectrum of compound **EDOT-C_{60}**	S-16
**Figure S13**. Closed [6,6]- and open [5,6]-aza-bridged adducts.	S-17
**Figure S14**. Photographic images of **PEDOT-C_{60}** films obtained by different number of polymerization cycles.	S-18
**Figure S15**. Microscopy images of (A) film PEDOT-C_{60} and (B) PEDOT-C_{60} with *S. aureus* biofilm (24 h incubation) under a bright field (100 × microscope objective, scale bar 2 μm).	S-19

Figure S16. Absorption spectra changes for the photobleaching of **PEDOT-C_{60}** surfaces after different irradiation times with visible light in PBS. S-20

Figure S17. Absorption spectra changes for the photooxidation of DMA photosensitized by (A) **PEDOT-C_{60}** and (B) fullerene C_{60} after different irradiation times. λ_{irr} = 455-800 nm. S-21

Figure S18. Absorption spectra changes of NBT after different irradiation time (A) film **PEDOT-C_{60}**, NBT and NADH (B) Fullerene C_{60} solution, NBT and NADH (C) NBT and NADH (D) film **PEDOT-C_{60}** and NTB (E) film **PEDOT-C_{60}** and NADH (F) film **PEDOT-C_{60}**, NBT and NADH dark control. [NBT] = 0.2 mM; [NADH] = 0.5 mM S-22

Figure S19. Emission spectra changes for the photooxidation of Trp photosensitized by (A) **PEDOT-C_{60}** and (B) fullerene C_{60} after different irradiation times. S-23

Experimental Procedures: General.

All chemicals were commercially acquired from Sigma-Aldrich and used without further purification. Dichloromethane (DMC) was dried by 4-hours reflux over P_2O_5 followed by distillation. Anhydrous toluene was prepared by reflux and distillation from Na/benzophenone ketyl. Reactions were run under an argon atmosphere with freshly anhydrous distilled solvents and employing oven-dried glassware, unless otherwise noted. The reactions were monitored by TLC (silica gel 60 GF254) run in different solvent mixtures. Flash column chromatographies were performed in silica gel 60 H (0,040-0,063 mM, 230-400 mesh ASTM, Merck) by gradient elution of mixture of n-hexane or toluene and increasing volumes of dichloromethane or ethyl acetate, respectively, under positive pressure of argon. Fourier transform Infrared (FT-IR) spectra were recorded on a Shimadzu Prestige 21 spectrophotometer as solid dispersions in KBr disks for solid samples or as thin films held between NaCl cells for oily samples. Nuclear magnetic resonance (NMR) spectra were performed in Insituto de Química de Rosario (IQUIR) on a FT-NMR Bruker Avance 300 spectrometer with Me_4Si as the internal standard and $CDCl_3$ as solvent. ^1H and ^{13}CNMR spectra were acquired at 300 and 75 MHz, respectively. Resonances of $CHCl_3$ in $CDCl_3$: δ 7.26 and 77.0 for ^1H and ^{13}C NMR, respectively. The magnitudes of the coupling constants (*J*) are given in Hertz. 2D-NMR experiments (COSY, HSQC, TOCSY and HMBC) were also recorded. Mass Spectra were taken with a Bruker micrO-TOF-QII (Bruker Daltonics, MA, USA) equipped with an ESI source (ESI-MS). Absorption spectra were carried out on a Shimadzu UV-2401PC spectrometer (Shimadzu Corporation, Tokyo, Japan). Photobleaching measurements were carried out on a UV-Visible Spectrophotometer Hewlett Packard-Diode Matrix 8453. Fluorescence spectra were performed on FluoroMax-4 spectrofluorometer (Horiba

Jobin Yvon Inc, Edison, NJ, USA). Cyclic voltammetry (CV) was carried out with a potentiostat-galvanostat Autolab (Electrochemical Instruments, Utrecht, The Netherlands). Scanning electron microscopy (SEM) images were obtained with a field emission scan-ning electron microscope FE-SEM (Σigma Zeiss, Oberkochen, Germany) with a thin Cr film on the sample surface and an acceleration voltage of 3 kV. Fluence rates were obtained with a Radiometer Laser Mate-Q (Coherent, Santa Clara, CA, USA).

The visible light source was a Novamat 130 AF (Braun Photo Technik, Nürnberg, Germany) slide projector equipped with a 150 W lamp. For 9,10-dimethylantracene (DMA) photolysis, a wavelength range between 455 and 800 nm was selected using an optical filter GG455 (fluence rate of 30 mW/cm^2). For visible light irradiation a wavelength range between 350 and 800 nm was selected by optical filters (fluence rate of 90 mW/cm^2)[i]. In all cases, spectral irradiated areas of the PSs were normalized in the range of irradiated wavelengths. Fluorescence images were obtained with a BIM500FL (Bioimager, Maple, ON, Canada) inverted epi-fluorescent microscope. Cell growth was measured with a Turner SP-830 spectrophotometer (Dubuque, IA, USA). Fluorescence images were obtained with a BIM500FL (Bioimager, Maple, ON, Canada) inverted epi-fluorescent microscope. Cell growth was measured with a Turner SP-830 spectrophotometer (Dubuque, IA, USA).

Controls and Statistical Analysis.

The experiments were repeated separately three times under the same conditions. Control measurements were also executed in the presence and absence of PEDO-C_{60} in the dark, and in the absence of polymer with cells irradiated. The unpaired t-test was used to establish the significance of differences between groups. Differences were considered

statistically significant with a confidence level of 95% ($p < 0.05$). Data were represented as the mean ± standard deviation of each group.

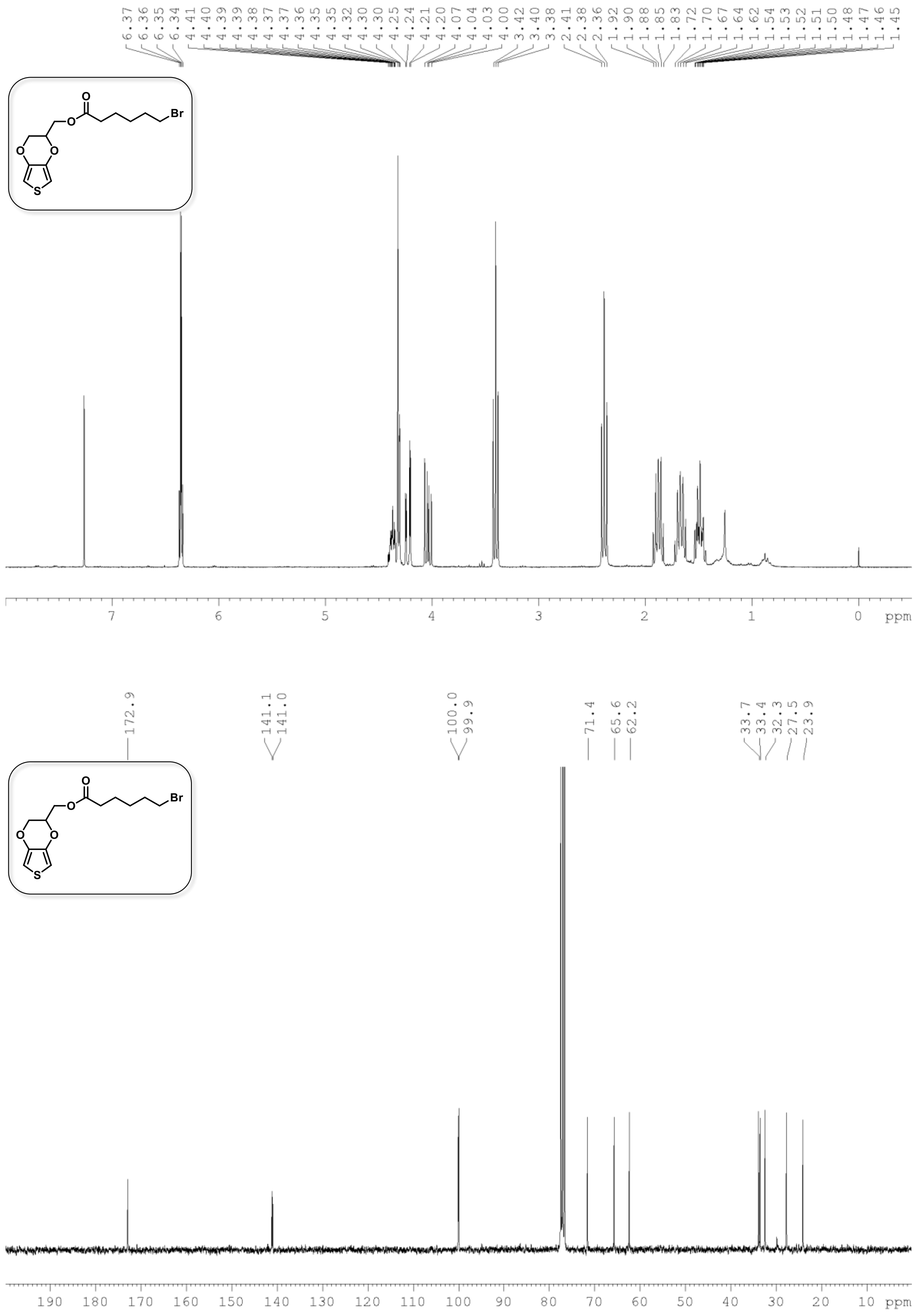

Figure S1. ^{1}H (top) and ^{13}C NMR (bottom) spectra of compound **EDOT-Br**.

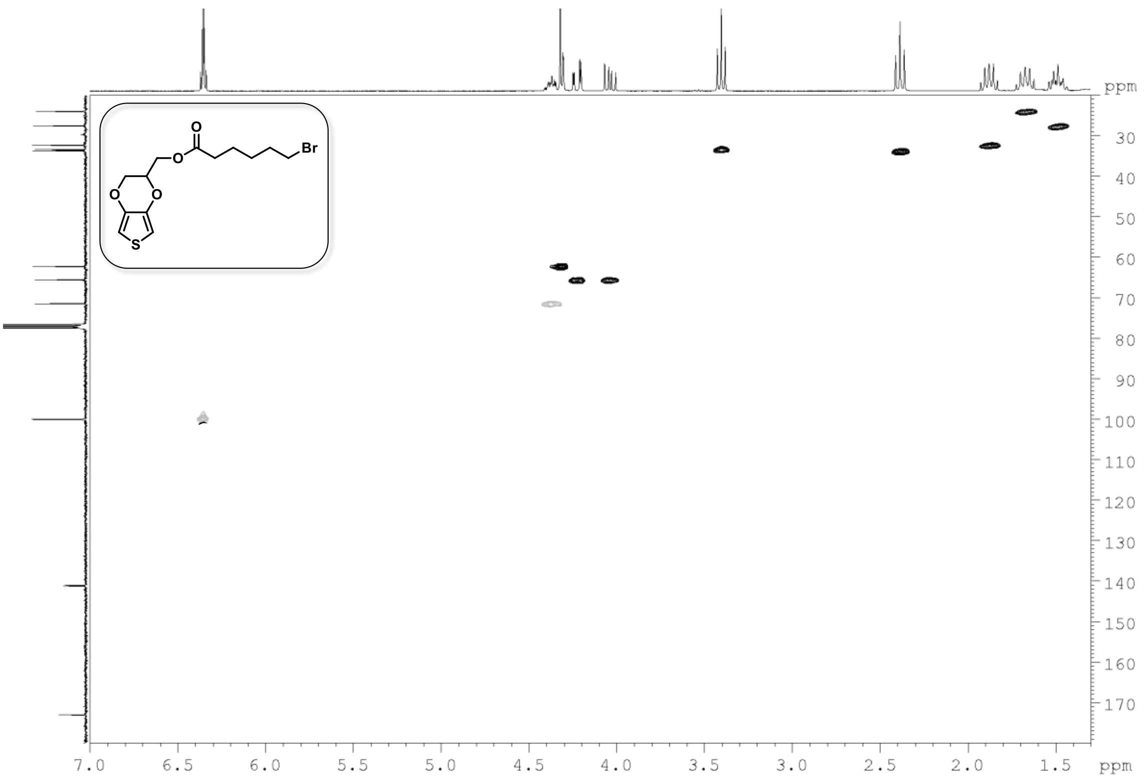

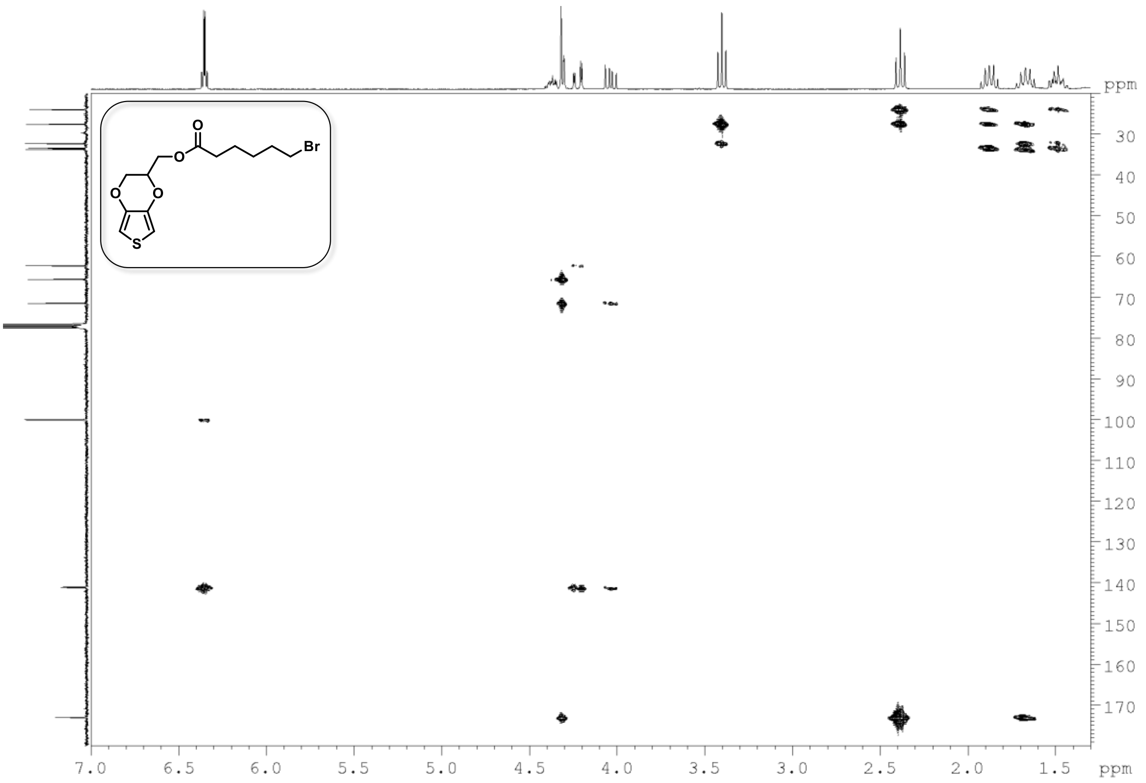

Figure S2. HSQC (top) and HMBC (bottom) NMR spectra of compound **EDOT-Br**.

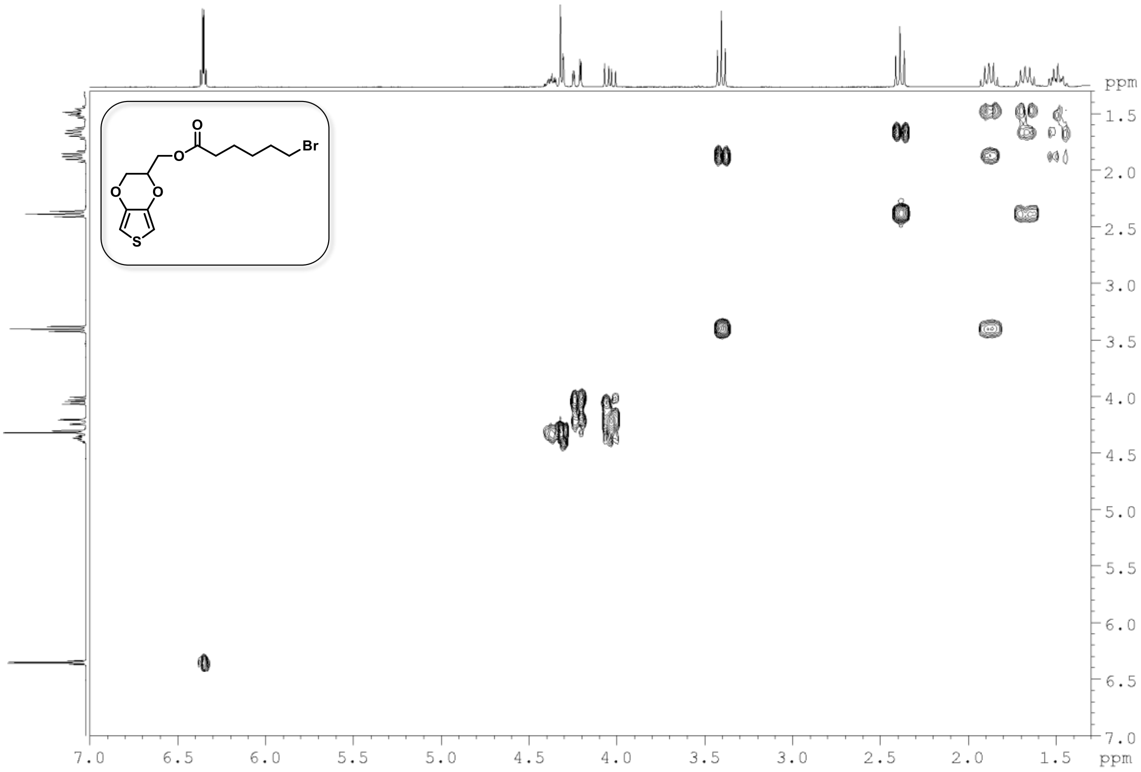

Figure S3. COSY NMR spectrum of compound **EDOT-Br**.

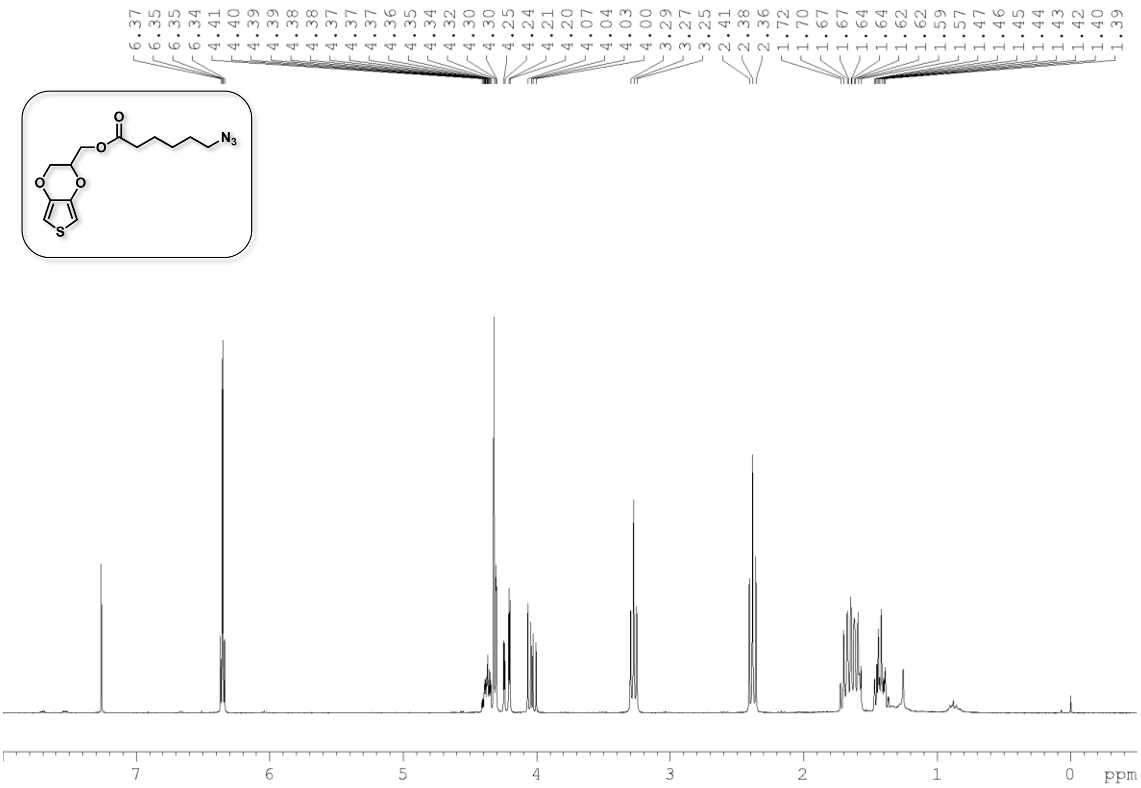

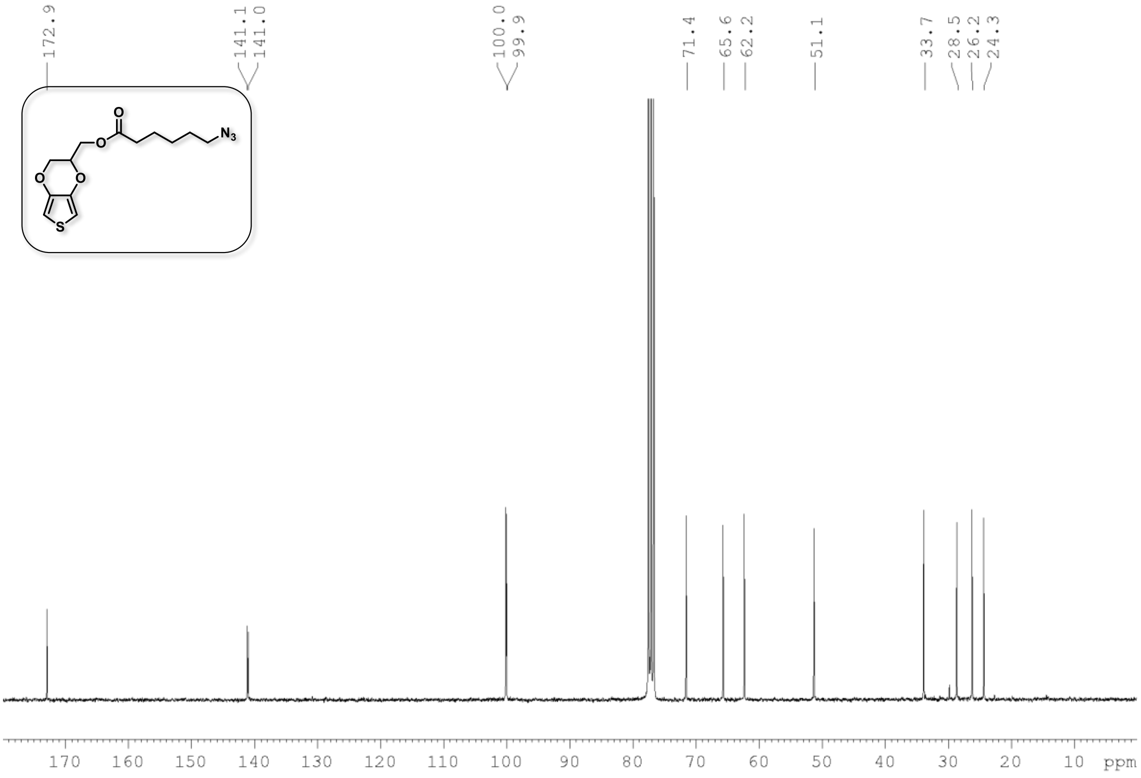

Figure S4. ^{1}H (top) and ^{13}C NMR (bottom) spectra of compound **EDOT-N$_3$**.

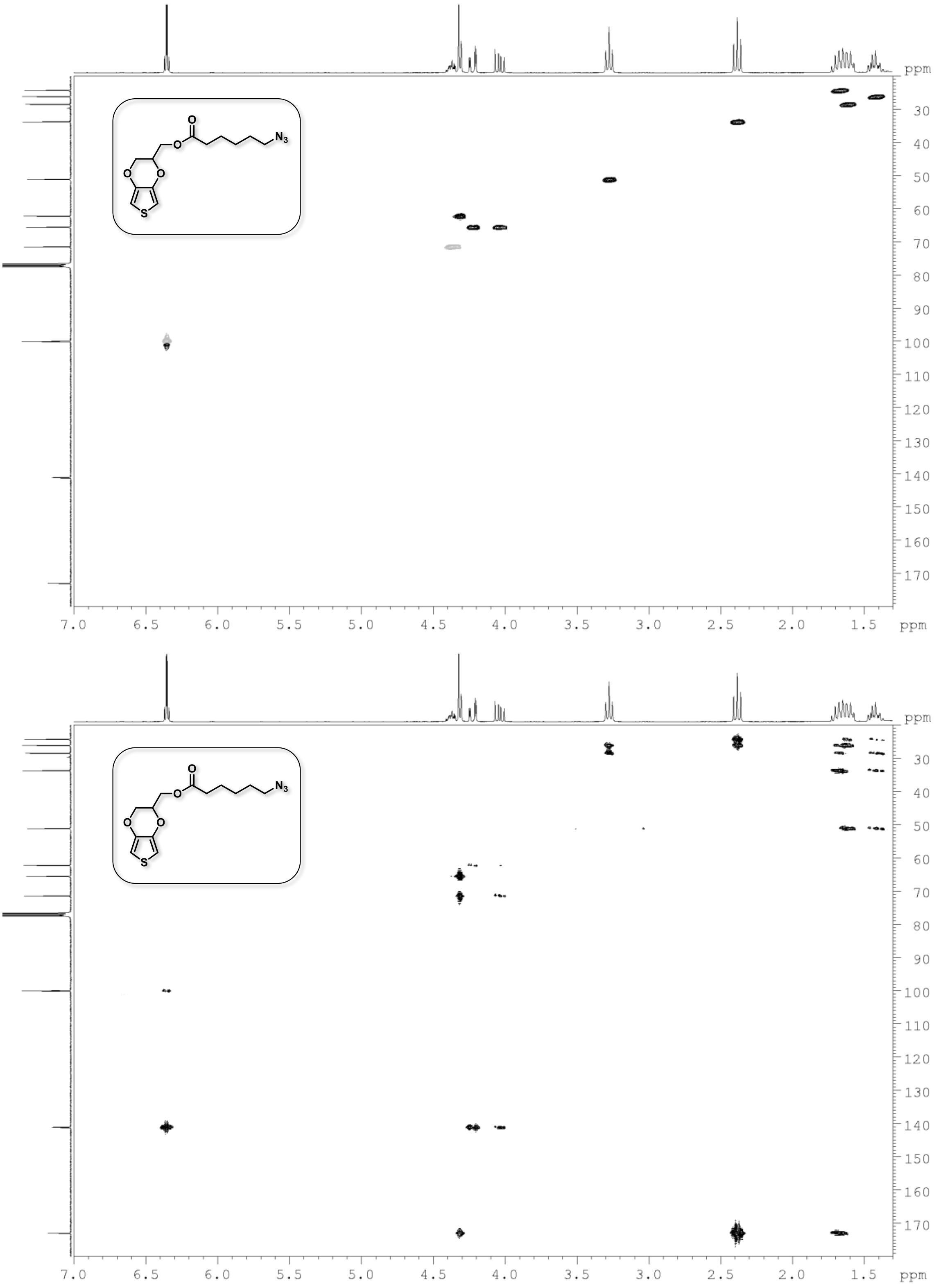

Figure S5. HSQC (top) and HMBC (bottom) NMR spectra of compound **EDOT-N$_3$**.

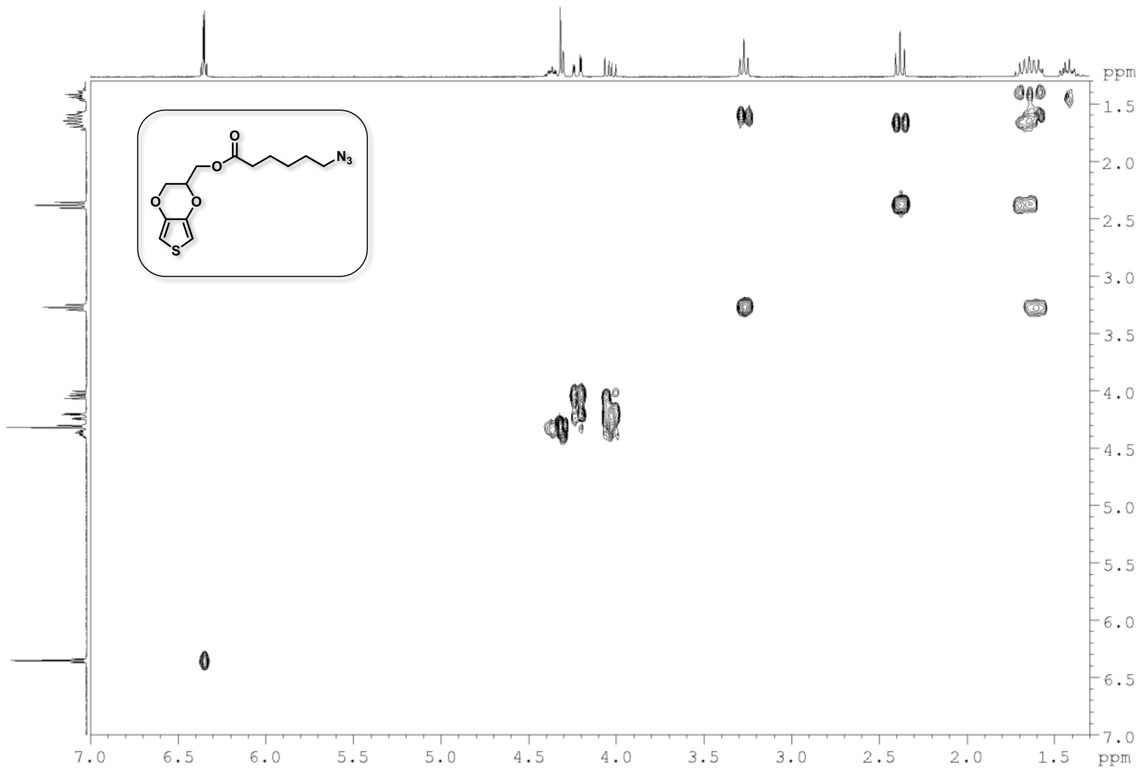

Figure S6. COSY NMR spectrum of compound **EDOT-N_3**.

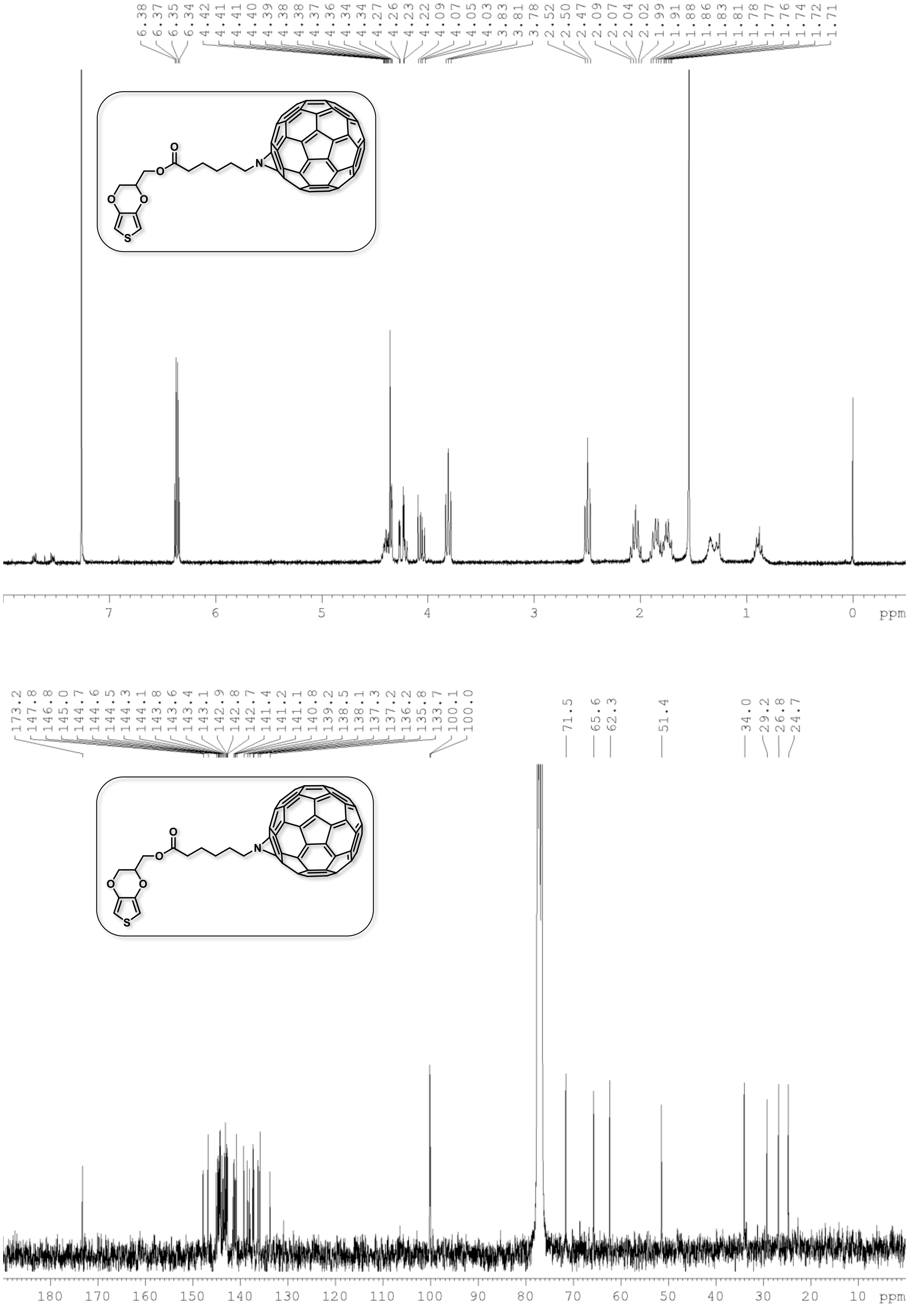

Figure S7. ^{1}H (top) and ^{13}C NMR (bottom) spectra of compound **EDOT-C$_{60}$**.

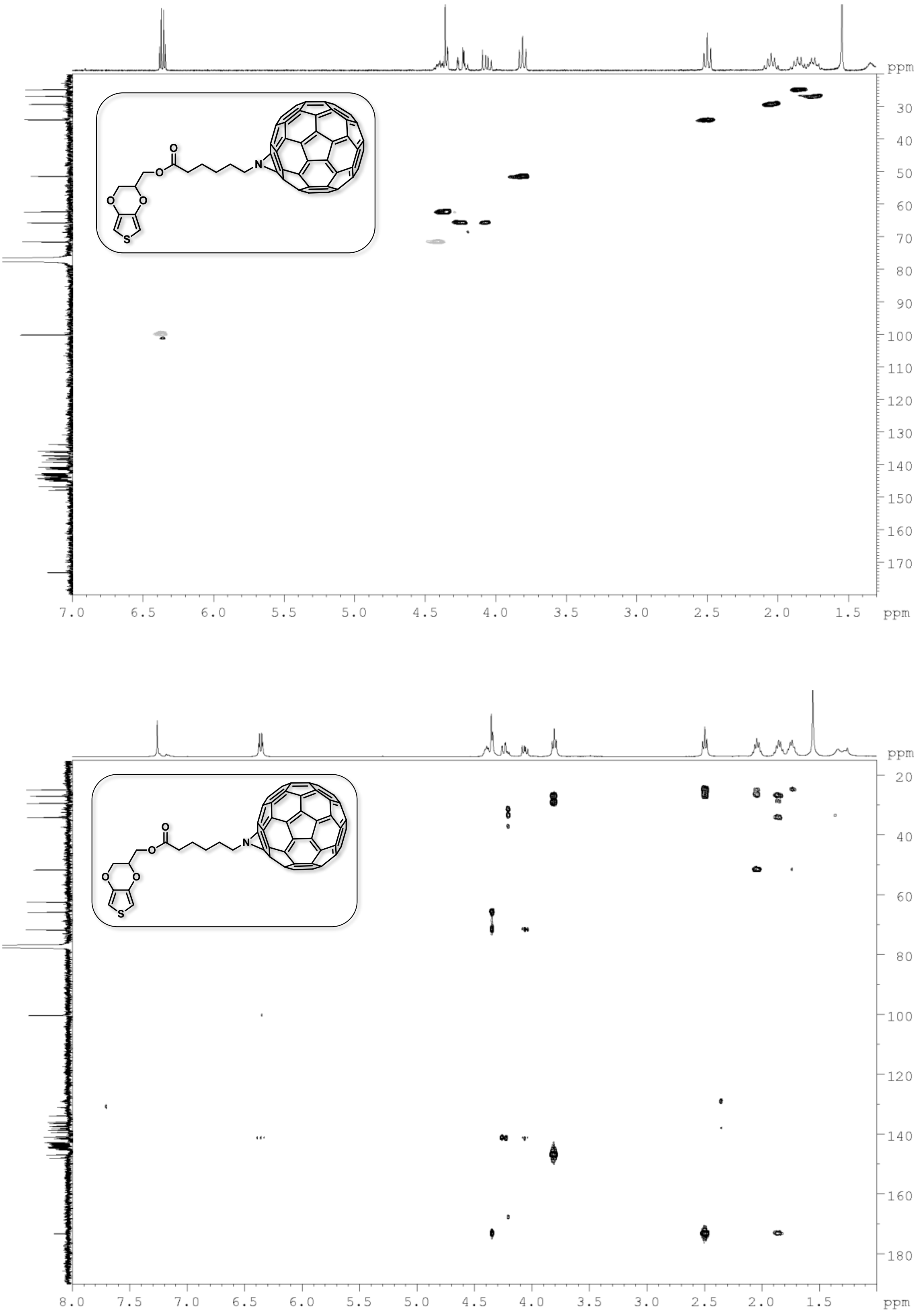

Figure S8. HSQC (top) and HMBC (bottom) NMR spectra of compound **EDOT-C_{60}**.

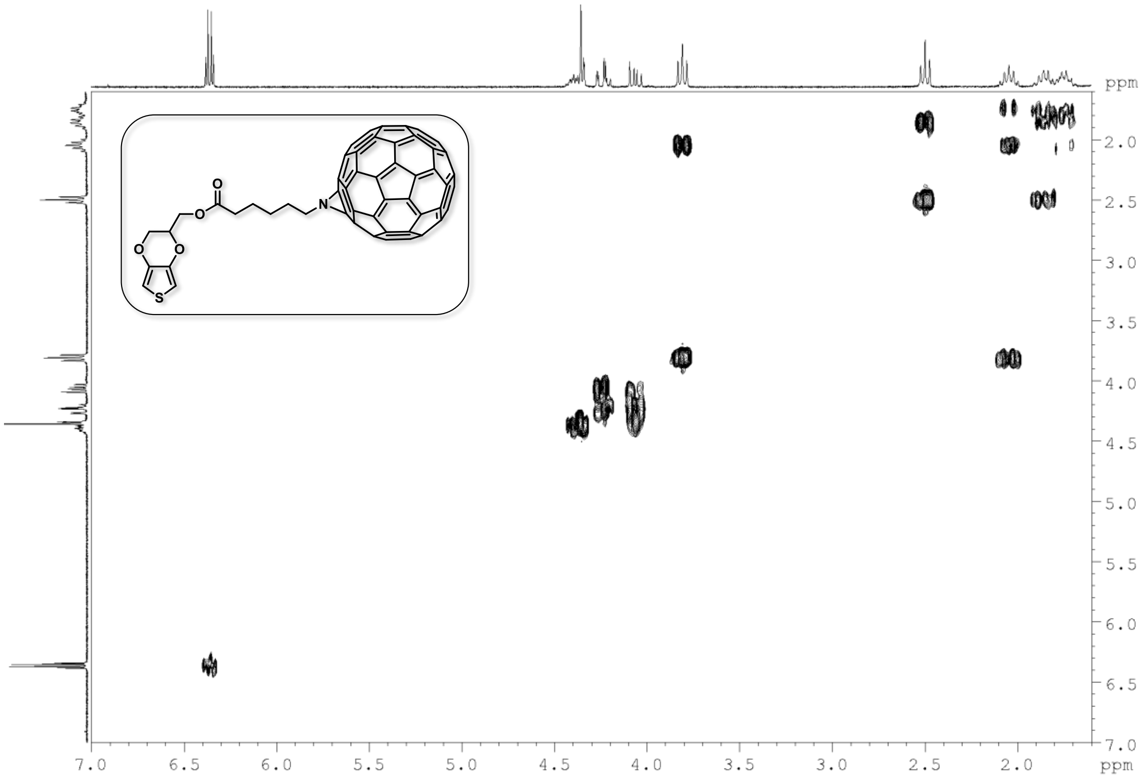

Figure S9. COSY NMR spectrum of compound **EDOT-C_{60}**.

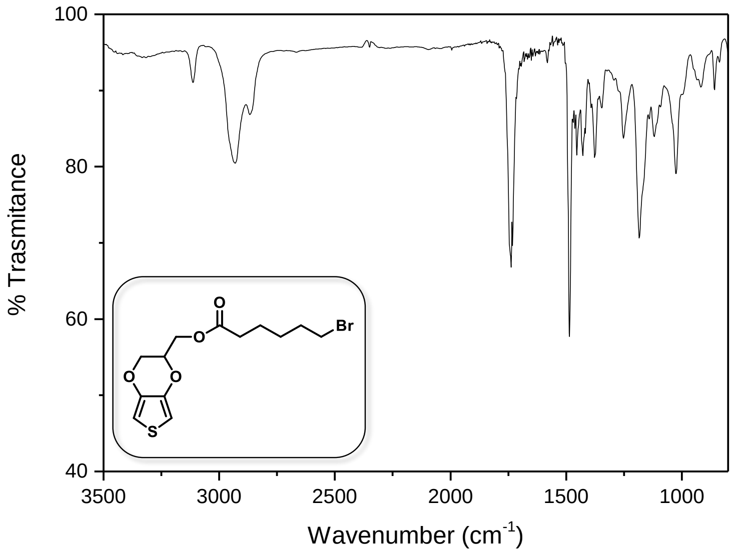

Figure S10. IR spectrum of compound **EDOT-Br**.

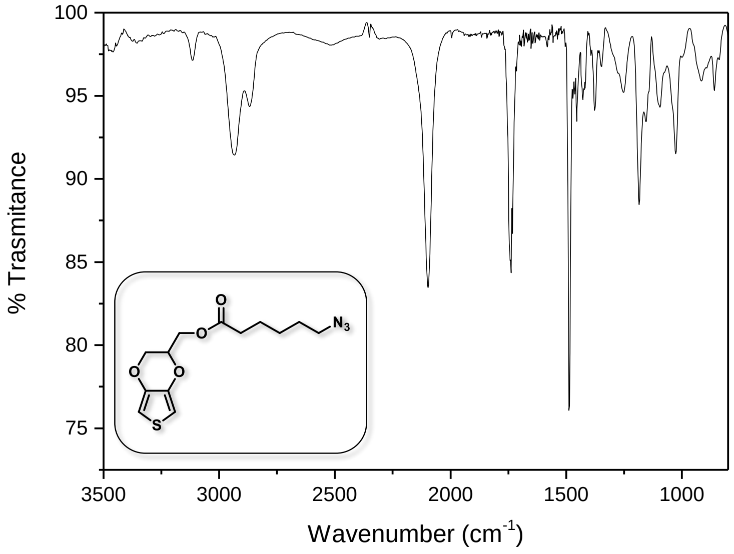

Figure S11. IR spectrum of compound **EDOT-N_3**.

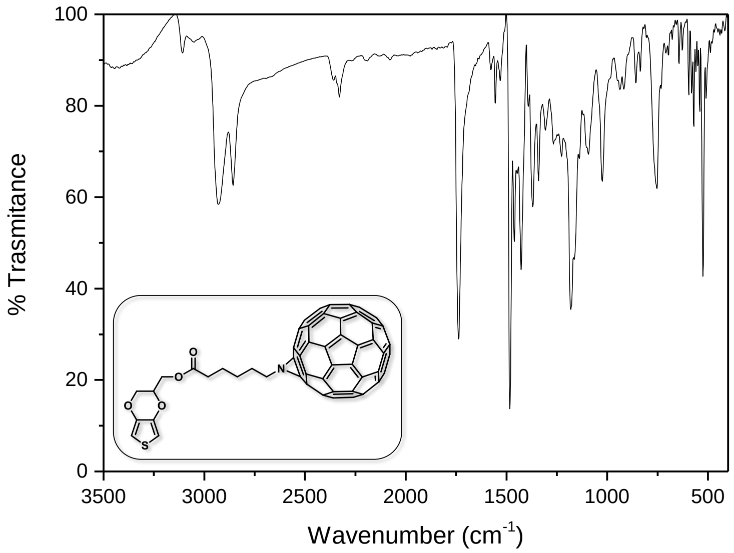

Figure S12. IR spectrum of compound **EDOT-C_{60}**.

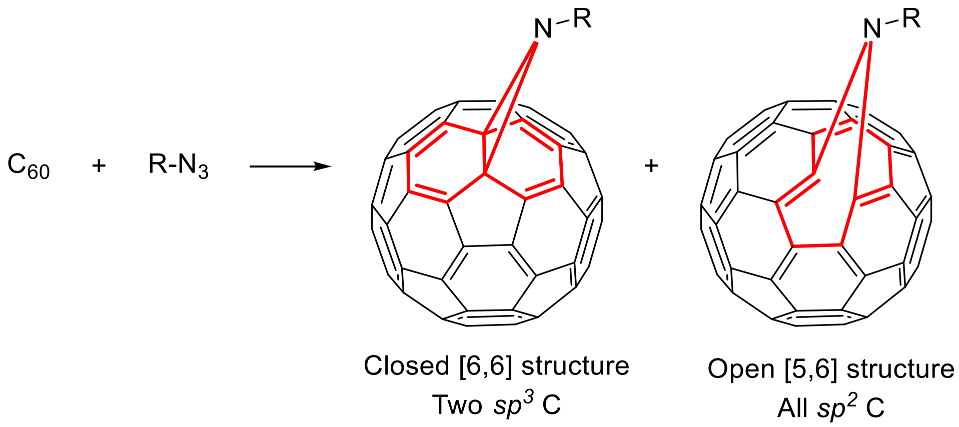

Figure S13. Closed [6,6]- and open [5,6]-aza-bridged adducts.

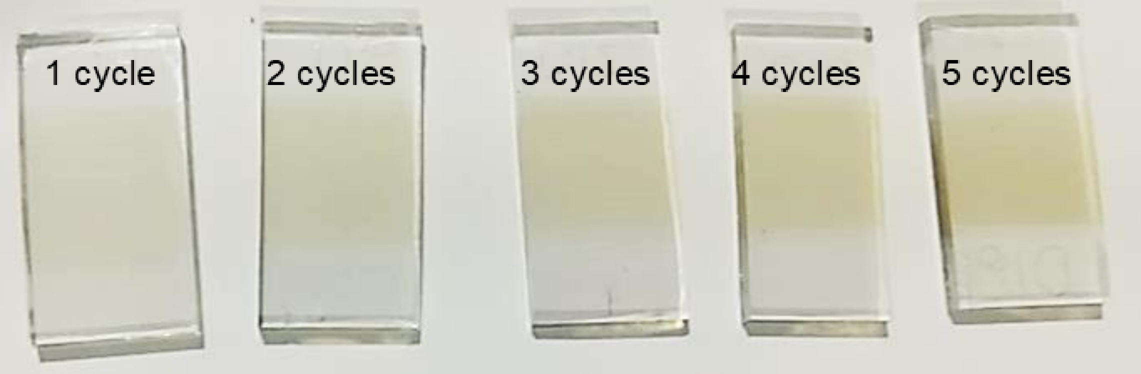

Figure S14. Photographic images of **PEDOT-C_{60}** films obtained by different number of polymerization cycles.

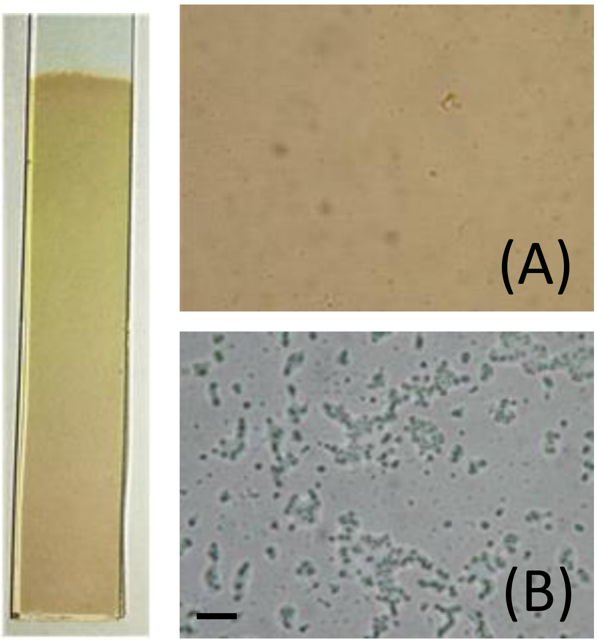

Figure S15. Microscopy images of **(A)** film **PEDOT-C_{60}** and **(B) PEDOT-C_{60}** with *S. aureus* biofilm (24 h incubation) under a bright field (100× microscope objective, scale bar 2 μm).

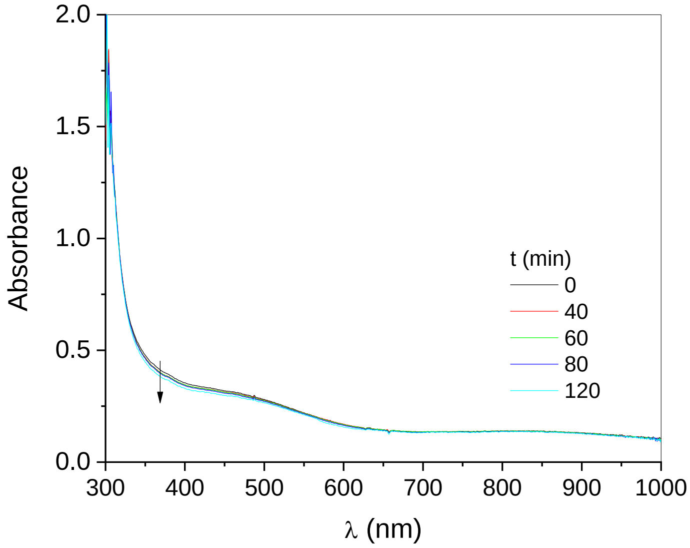

Figure S16. Absorption spectra changes for the photobleaching of PEDOT-C60 surfaces after different irradiation times with visible light in air.

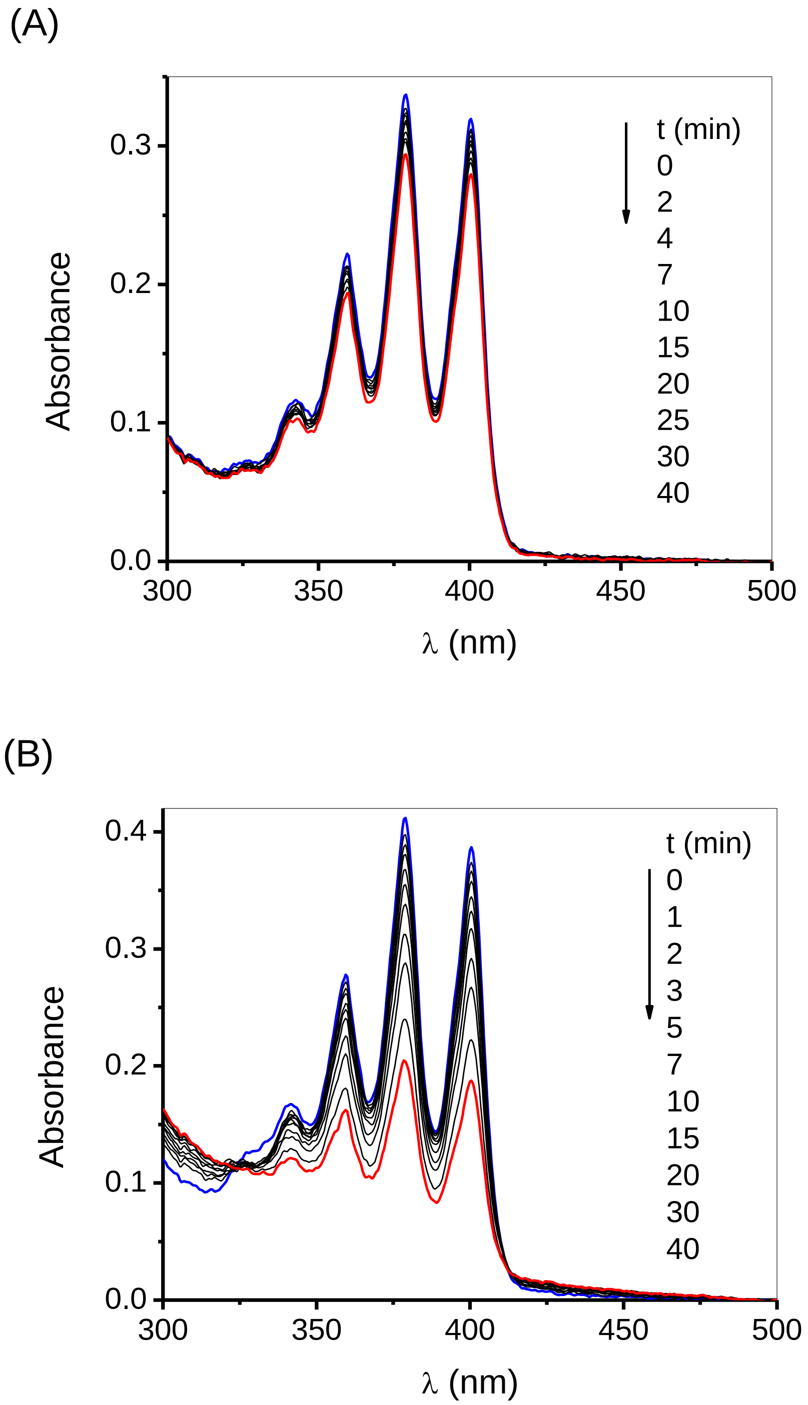

Figure S17. Absorption spectra changes for the photooxidation of DMA photosensitized by (A) **PEDOT-C_{60}** and (B) fullerene C_{60} after different irradiation times. λ_{irr} = 455-800 nm.

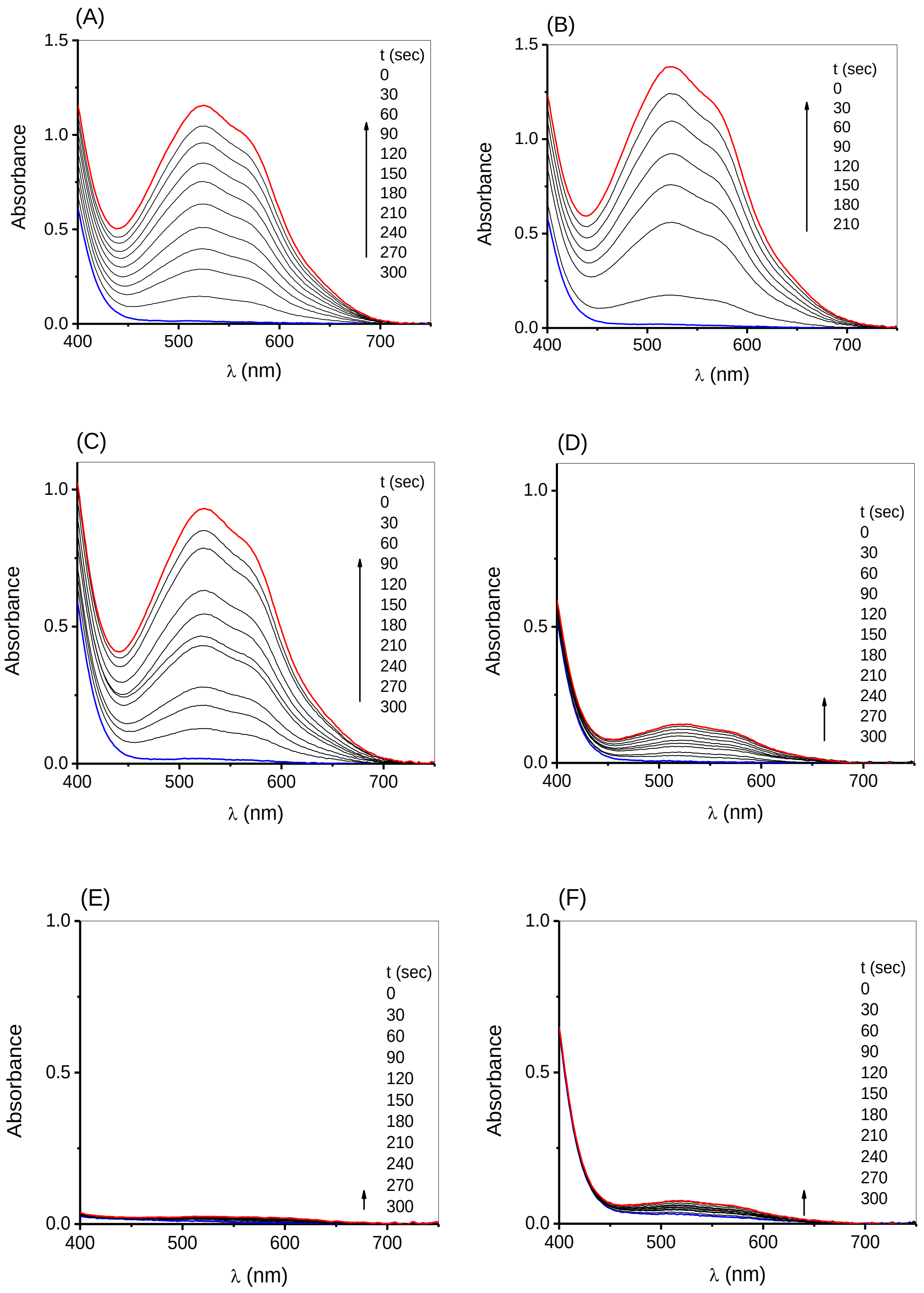

Figure S18. Absorption spectra changes of NBT after different irradiation time **(A)** film **PEDOT-C$_{60}$**, NBT and NADH **(B)** Fullerene C$_{60}$ solution, NBT and NADH **(C)** NBT and NADH **(D)** film **PEDOT-C$_{60}$** and NTB **(E)** film **PEDOT-C$_{60}$** and NADH **(F)** film **PEDOT-C$_{60}$**, NBT and NADH dark control. [NBT] = 0.2 mM; [NADH] = 0.5 mM.

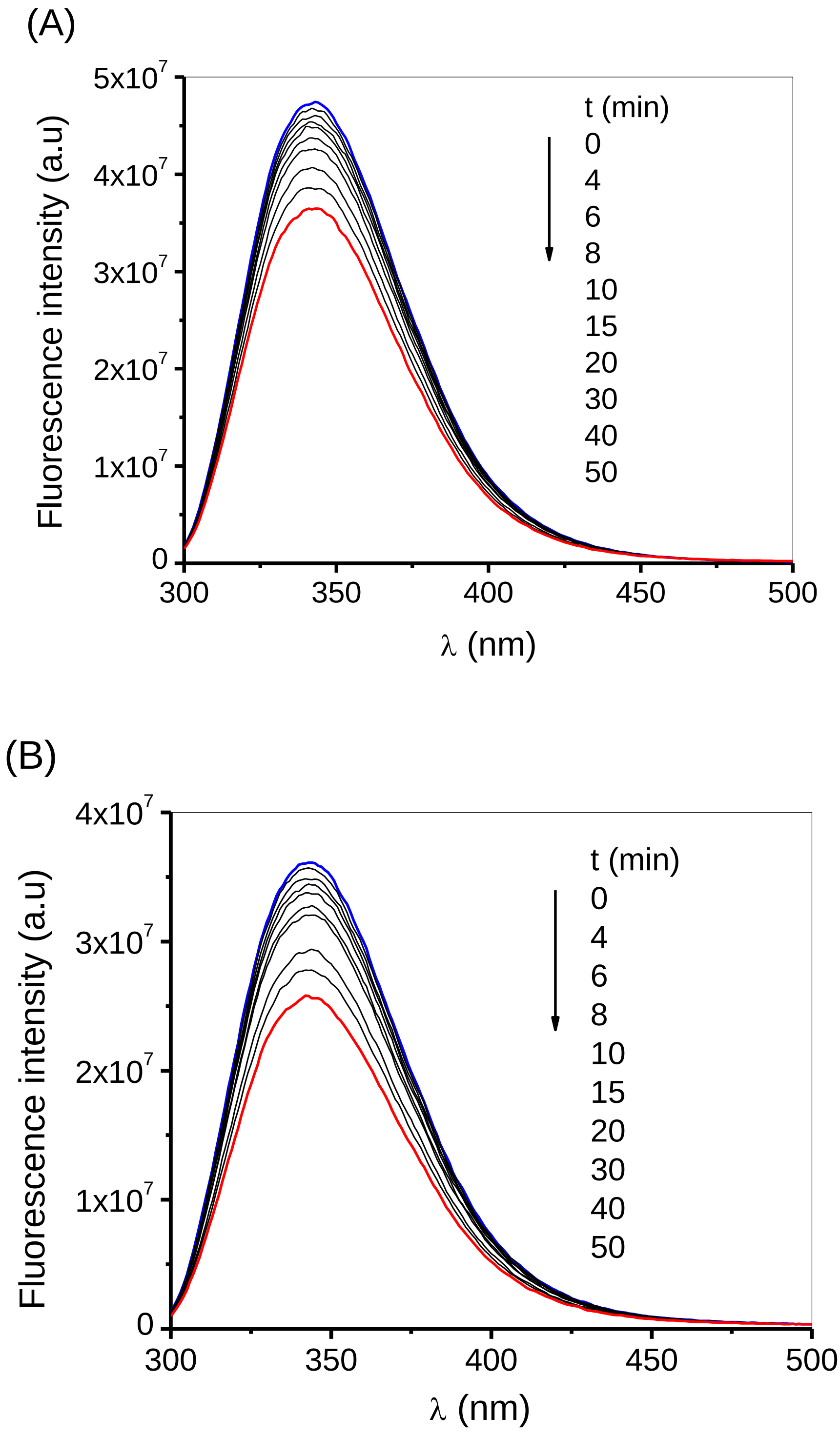

Figure S19. Emission spectra changes for the photooxidation of Trp photosensitized by (A) **PEDOT-C_{60}** and (B) fullerene C_{60} after different irradiation times.

i M. B. Ballatore, M. E. Milanesio, H. Fujita, J. S. Lindsey, E. N. Durantini, *J. Biophotonics.*, 2020, **13**, e201960061.
